# Genome-Wide Identification and Functional Analyses of the CRK Gene Family in Cotton Reveals *GbCRK18* Confers Verticillium Wilt Resistance in *Gossypium barbadense*

**DOI:** 10.3389/fpls.2018.01266

**Published:** 2018-09-11

**Authors:** Ting-Gang Li, Dan-Dan Zhang, Lei Zhou, Zhi-Qiang Kong, Adamu S. Hussaini, Dan Wang, Jun-Jiao Li, Dylan P. G. Short, Nikhilesh Dhar, Steven J. Klosterman, Bao-Li Wang, Chun-Mei Yin, Krishna V. Subbarao, Jie-Yin Chen, Xiao-Feng Dai

**Affiliations:** ^1^Laboratory of Cotton Disease, Institute of Food Science and Technology, Chinese Academy of Agricultural Sciences, Beijing, China; ^2^Department of Plant Pathology, University of California, Davis, Davis, CA, United States; ^3^U.S. Agricultural Research Station, Salinas, CA, United States; ^4^Crop Improvement and Protection Research Unit, United States Department of Agriculture, Agricultural Research Service, Salinas, CA, United States

**Keywords:** cotton, Verticillium wilt, cysteine-rich receptor-like kinases (*CRKs*), expression profiling, defense response

## Abstract

Cysteine-rich receptor-like kinases (*CRKs*) are a large subfamily of plant receptor-like kinases that play a critical role in disease resistance in plants. However, knowledge about the *CRK* gene family in cotton and its function against Verticillium wilt (VW), a destructive disease caused by *Verticillium dahliae* that significantly reduces cotton yields is lacking. In this study, we identified a total of 30 typical *CRKs* in a *Gossypium barbadense* genome (*GbCRKs*). Eleven of these (>30%) are located on the A06 and D06 chromosomes, and 18 consisted of 9 paralogous pairs encoded in the A and D subgenomes. Phylogenetic analysis showed that the *GbCRKs* could be classified into four broad groups, the expansion of which has probably been driven by tandem duplication. Gene expression profiling of the *GbCRKs* in resistant and susceptible cotton cultivars revealed that a phylogenetic cluster of nine of the *GbCRK* genes were up-regulated in response to *V. dahliae* infection. Virus-induced gene silencing of each of these nine *GbCRKs* independently revealed that the silencing of *GbCRK18* was sufficient to compromise VW resistance in *G. barbadense*. GbCRK18 expression could be induced by *V. dahliae* infection or jasmonic acid, and displayed plasma membrane localization. Therefore, our expression analyses indicated that the *CRK* gene family is differentially regulated in response to Verticillium infection, while gene silencing experiments revealed that *GbCRK18* in particular confers VW resistance in *G. barbadense*.

## Introduction

Plants are often infected by various pathogens including bacteria, fungi, oomycetes, and nematodes, but unlike animals, plants lack adaptive/acquired immune system, and rely entirely on innate immunity to resist numerous potential pathogens in the environment (Jones and Dangl, 2006; Boller and Felix, 2009; Zhou et al., 2017). To counteract pathogen invasion, plants have evolved different types of resistance proteins which activate immune responses and restrict pathogen proliferation (Chisholm et al., 2006; Jones and Dangl, 2006; Dodds and Rathjen, 2010), and these proteins can be classified into different types based primarily upon the presence of specific conserved structural motifs (Martin et al., 2003; Kruijt et al., 2005; Joshi and Nayak, 2011). Among these, a diverse array of plasma membrane-bound receptors are employed by plants to perceive endogenous and exogenous signals for regulation of immunity, and these cell surface receptors include receptor-like kinases (RLKs) and receptor-like proteins (RLPs) that harbor different extracellular domains for perception of distinct ligands (He et al., 2018).

The plant RLKs were first discovered in maize (Walker and Zhang, 1990) and represent the most abundant molecular recognition receptors found in plants. More than 600 genes encoding protein-like receptor kinases have been found in *Arabidopsis*, accounting for 2.5% of its gene pool (Shiu and Bleecker, 2001). Nearly 1300 genes in rice encode RLKs (Shiu et al., 2004). The typical RLK contains a signal peptide, a variable extracellular domain, a transmembrane domain, and an intracellular kinase domain (Van et al., 1994). RLKs are divided into different families according to their extracellular ligand binding domain, such as leucine-rich repeats (LRRs), lectin, lysine motif (LysM), and wall-associated kinases (WAK) (Tör et al., 2009). In general, the *N*-terminal extracellular region (the ectodomain) of the RLKs extends into the apoplast where it perceives the signals from the pathogen, whereas the C-terminal kinase domain resides within the cytoplasm and relays the perceived signals into the intracellular environment (Kimura et al., 2017).

Cysteine-rich receptor-like kinases (CRKs), also called domain of unknown function 26 (DUF26) RLKs, have a role in disease resistance and plant cell death (Chen et al., 2003). Extracellular domains of CRKs contain two copies of the DUF26 motif and have conserved Cys residues within the sequence C-X8-C-X2-C. The C-X8-C-X2-C domain is a novel motif structurally distinct from the Cys-rich region of S-locus glycoproteins or S-domain RLKs (Chen, 2001). These conserved Cys residues may participate in the formation of the three-dimensional structure of the protein through disulfide bonds or form zinc finger motifs, as in many DNA-binding transcription factors. Both disulfide bonds and zinc fingers are known to mediate protein–protein interactions, a critical step in the activation of many receptors, including those with protein kinase domains upon ligand binding (Hardie, 1999).

Cysteine-rich receptor-like kinases involved in plant defense responses have been well-studied in *Arabidopsis* and other model systems: overexpression of *Arabidopsis CRK5*, *CRK6*, *CRK36*, and *CRK45* can significantly enhance resistance against *Pseudomonas syringae* pv. tomato DC3000, via rapid expression of the defense genes and ROS production (Chen et al., 2003; Zhang X. et al., 2013; Yeh et al., 2015); *CRK4*, *CRK19*, and *CRK20* can be significantly induced by salicylic acid (SA) and pathogen infection (Chen et al., 2004; Ederli et al., 2011); *CRK6* and *CRK7* have an important role in extracellular ROS signaling (Idänheimo et al., 2014); up-regulation of *CRK13* leads to the hypersensitive response associated with cell death, and induces defense against pathogens by causing increased accumulation of SA (Acharya et al., 2007); *CRK28* and *CRK29* can be induced in response to flagellin; and overexpression of *CRK28* in *Nicotiana benthamiana* that results in cell death (Yadeta et al., 2017). Aside from these examples, the wheat *CRK TaCRK1* is a resistance factor against *Rhizoctonia cerealis* (Yang et al., 2013). In barley, a *CRK* gene designated as *HvCRK1*, which encodes an endoplasmic reticulum-associated protein involved in the negative regulation of basal resistance in barley against *Blumeria graminis* f.sp. *hordei* (Rayapuram et al., 2012).

Verticillium wilt (VW) is caused by soil-borne *Verticillium dahliae*, a fungus with a broad host range of over 400 plant species, and this pathogen can survive in soil for many years (Tao et al., 2016). In cotton, VW is a devastating disease that results in major losses in yield and boll quality (Li et al., 2011). Improving host resistance is considered the most optimal method to manage VW, which entails identifying VW resistance genes in cotton germplasm and incorporating them into elite cotton varieties. To date, several genes have been characterized that contribute to defense responses against VW, including *GbCAD1* and *GbSSI2* (Gao et al., 2013), *GbRLK* (Zhao et al., 2015), *GbSTK* (Zhang Y. et al., 2013), *GbTLP1* (Munis et al., 2010), *GbSBT1* (Duan et al., 2016), *GhPAO* (Mo et al., 2015), *GbNRX1* (Li et al., 2016), *GbRVd* (Yang et al., 2016), and *GbVe/GbVe1*/*Gbvdr5* (Zhang et al., 2011; Zhang B. et al., 2012; Yang et al., 2015).

Currently, genomic analysis is an effective means of transferring knowledge from one taxon to another (Zhang Y. et al., 2012). At present, the genomes of diploid and allotetraploid cottons have been released and can facilitate our understanding of the expansion and functions of the *CRK* family genes in cotton. Except for one study (Zhang et al., 2018), no CRKs involved in disease resistance have been reported in cotton. The allotetraploid cotton *G. barbadense* is generally recognized as a VW-resistant species that contains abundant resistance gene resources (Zhang J. et al., 2012). The objectives of this current study were to: (1) identify members of the *CRK* gene family in *G. barbadense* genome; (2) elucidate the structure and characteristics of the *G. barbadense CRK* gene family; (3) investigate the candidate *CRK* genes involved in VW in cotton; (4) explore the relationships between plant hormones and defense responses mediated by the candidate CRK genes.

## Materials and Methods

### Identification of the Cotton *CRK* Gene Family

An available genomic database of *G. barbadense* was downloaded from http://database.chgc.sh.cn/cotton/index.html; and for the other three cotton species, *Gossypium arboretum*, *Gossypium raimondii*, and *Gossypium hirsutum*, data were all downloaded from https://www.cottongen.org/; data for *Arabidopsis thaliana*, *Zea mays*, and *Oryza sativa* were downloaded from http://www.plantgdb.org/. The Pfam protein family databases^1^ with the Stress-antifung (PF01657) and Pkinase (PF00069) (Finn et al., 2014) domains were used with the HMMER software version 3.0 (Eddy, 2011) to identify the *CRK* genes in these seven species. Putative gene sequences were further verified using the SMART database (Letunic and Bork, 2018) and InterproScan (Hunter et al., 2012). Stress-antifung domains of the *CRK* proteins were investigated statistically using MEME website^2^. Prediction of signal peptides and signal peptide cleavage sites of putative extracellular proteins were conducted using SignalP (version 4.1; *D*-Score cut-off set to 0.500) (Petersen et al., 2011). The CRK proteins with signal peptides were analyzed for the presence of transmembrane domains using TMHMM 2.0 (Krogh et al., 2001).

### Mapping, Phylogenetic Tree Construction, and Structural Analysis

MapInspect^3^ was used to analyze the distribution of the *CRK* genes in the *G. barbadense* genome. The multiple sequence alignments of CRK proteins from seven species were carried out using ClustalX (version 1.83). A phylogenetic tree was built by MEGA 6 software^4^ using the neighbor-joining (NJ) method (bootstrap value was set 1000). The exon/intron structures were analyzed by aligning the genomic DNA sequences with their corresponding coding sequences using the online Gene Structure Display Server (GSDS)^5^. The similarity of 30 *CRK* genes was then compared and analyzed using local BLAST analysis.

### Plant Materials and Treatments

Seedlings of *G. barbadense* cv. Hai 7124 and *G. barbadense* cv. GZ 57 were used for gene expression analyses under different stress treatments. Plants were grown in the same controlled environment chambers under 16 h light/8 h dark cycle at 28°C for 2 weeks. Seedlings of cv. Hai 7124 and cv. GZ 57, which exhibit, respectively, resistance and susceptibility to *V. dahliae*, were inoculated with the *V. dahliae* isolate Vd991 using the root dip-inoculation method (Wang et al., 2011). Vd991, a highly aggressive defoliating strain of *V. dahliae*, was cultured in complete medium (CM) at 25°C for 5 days. The concentration of conidia was adjusted to 5 × 10^6^ conidia/ml using deionized water and subsequently used for inoculation of seedlings (Li et al., 2018). The seedling roots were harvested with three replicates at eight time intervals (0, 2, 6, 12, 24, 48, 72, and 120 h) after Vd991 inoculation. Each replicate consisted of five seedlings, which were quickly frozen in liquid nitrogen and stored at -80°C for RNA extraction.

Leaves of 3-week-old cotton seedlings were sprayed with 10 mM SA, 10 mM ethephon (ETH), 10 mM methyljasmonate (MeJA), or 100 mM abscisic acid (ABA), respectively. Sterile distilled water served as a solvent control, and the leaves were collected at seven time points (0, 2, 6, 12, 24, 48, and 72 h) for RNA extraction. Three plants per replicate were harvested at each time point and stored at -80°C after flash-freezing in liquid nitrogen.

*GbCRK18* was silenced in *G. barbadense* cv. Hai 7124 through virus-induced gene silencing (VIGS), and the expression level of *GbCRK18* was assessed in the silenced plants to determine the efficiency of silencing. After determining the success of silencing protocol, wild-type and silenced plants were inoculated with *V. dahliae* Vd991 using a root dip-inoculation at 5 × 10^6^ conidia/ml. Twelve hours after inoculation, cotton roots were harvested and washed with water for RNA extraction to detect the expression of marker resistance genes involved in the JA pathway. Empty vector pTRV2-silenced plants served as controls.

### RNA Isolation and Analyses of Gene Expression

Total RNA was extracted from cotton seedling roots using a Plant RNA Purification Kit (Tiangen, Beijing, China). The cDNA was prepared using M-MLV reverse transcriptase and reverse transcription (RT)-qPCR analyses were conducted using the SYBR Premix Ex Taq kit (TaKaRa, Japan) with a QuantStudio 6 Flex qPCR System (Applied Biosystems, Foster City, CA, United States). Primers used for RT-qPCR amplification are listed in **Supplementary Table S1** and were designed to avoid conserved regions within the members of the *CRK* family. The cotton *18S* gene was used as an internal control to normalize the variance among samples. RT-qPCR conditions consisted of an initial denaturation step at 95°C for 10 min, followed by 40 cycles of denaturation at 95°C for 15 s, annealing at 60°C for 30 s, and extension at 72°C for 20 s. Relative expression levels were evaluated using the 2^-ΔΔCT^ method (Livak and Schmittgen, 2001).

### Cloning of the *GbCRK18* Gene

Primers were designed according to the full open-reading frame (ORF) of the gene GOBAR_DD09713 in the *G. barbadense* reference genome (Liu et al., 2015; **Supplementary Table S1**). These primers were used to amplify the target fragment from *G. barbadense* cDNA. PCR conditions consisted of an initial 95°C denaturation step for 10 min, followed by 39 cycles at 95°C for 45 s, 55°C for 45 s, and 72°C for 2.5 min. All PCR products were cloned into the pGEM-T-Easy vector (Promega, Madison, WI, United States) and transformed into *Escherichia coli* DH5α. At least six clones were arbitrarily selected and sequenced.

### Functional Characterization of Candidate *CRK* Genes via Virus-Induced Gene Silencing (VIGS) Analysis

For the VIGS assays, one fragment from each gene, *GbCRK02*, *GbCRK03*, *GbCRK06*, *GbCRK07*, *GbCRK08*, *GbCRK18*, *GbCRK19*, *GbCRK22*, and *GbCRK23*, was amplified from *G. barbadense* cDNA and integrated into the vector pTRV2 to construct pTRV2:*CRK.n*, which was introduced into *Agrobacterium tumefaciens* GV3101. Primers used for construction of the above VIGS vectors are listed in **Supplementary Table S1**. *Agrobacterium* strains harboring pTRV2:*CRK.n* plasmid were combined with *Agrobacterium* strains harboring the pTRV1 vector were mixed in a 1:1 ratio and co-infiltrated into the cotyledons of 2-week-old resistant cv. Hai 7124 plants, with cv. GZ 57 serving as a susceptible control. Control vector pTRV2:*CLA1* was used to evaluate the effectiveness of VIGS since effective silencing of this gene leads to the development of white leaves. Two weeks later, white leaves were observed on plants in which the *CLA1* gene had been targeted and silenced by VIGS, then plantlets were subjected to *V. dahliae* inoculation with 10 ml of conidial suspension (5 × 10^6^ conidia/ml), and VW symptoms were investigated at 3 weeks post-inoculation. Seedling shoots were cut to investigate the vascular wilt symptom under a microscope (Liu et al., 2013; Chen et al., 2017). For fungal biomass quantification in planta, stems of three inoculated plants were harvested at 20 days post inoculation. qPCR was conducted with template genomic DNA isolated from the samples using the SYBR Premix Ex Taq kit (Takara, Japan). *V. dahliae* elongation factor 1-α (*EF-1α*) and cotton *18S* genes were used to quantify fungal colonization by qPCR (Santhanam et al., 2013).

### Subcellular Localization Analysis of the *CRK18*

To examine the subcellular localization of *Gb*CRK18, we inserted the full-length *GbCRK18* coding region into the pBin-*GFP4* vector to generate a C-terminal fusion with the *GFP* gene under the control of a cauliflower mosaic virus (CaMV) 35S promoter (p35S:*GbCRK18*). Plasmids harboring *GFP* alone (empty vector, p35S:*GFP*) were used as controls. The p35S:*GFP* control and p35S:*GbCRK18* vectors fused to *GFP* were transiently expressed in tobacco epidermal cells by infiltration with *Agrobacterium*. Subcellular localization of the p35S:*GFP* and p35S:*GbCRK18* fusion proteins was observed by laser scanning confocal microscopy (LSMT-PMT) with excitation and emission wave lengths of 488 and 510 nm, respectively.

## Results

### Genome-Wide Identification of *CRK* Gene Family

**FIGURE 1:**
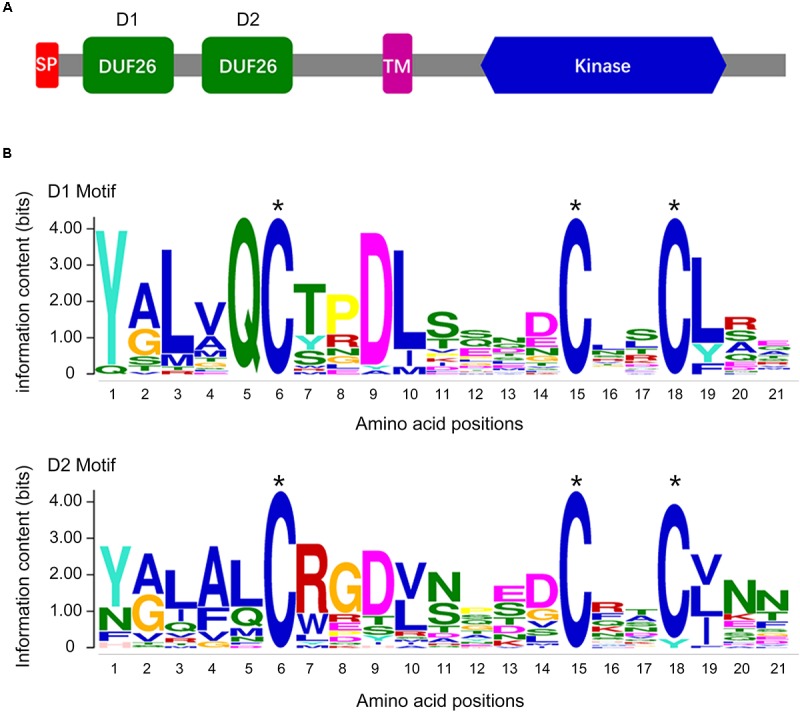
The DUF26 domain organization and the conserved structure of the *CRK* family. **(A)** Conserved structure of the *CRK* family: a signal peptide (SP), two stress-antifung domains (DUF26), a transmembrane domain (TM), and an intracellular kinase domain (Kinase); **(B)** conserved DUF26 domain.

To evaluate the *CRK* gene family in a cotton genome, we performed an HMMER alignment search on the proteome of the *G. barbadense* genome with the hidden Markov model (HMM) profile of the Stress-antifung domain (PF01657) and Pkinase domain (PF00069). Manual selection resulted in the identification of 63 candidate *CRK* family members (**Supplementary Table S2**), and all of them were further subjected to domain analysis using SMART and InterproScan, which defined a set of typical CRK proteins that included a signal peptide, two stress-antifung domains, one transmembrane domain (TM), and one Pkinase domain (**Figure 1A**). A total of 30 *CRK* genes (*GbCRK*) were identified in the *G*. *barbadense* genome, which were numbered from *GbCRK1* to *GbCRK30* according to their localization on chromosomes (**Table 1**). The peptides encoded by *GbCRKs* consisted of 521–1817 amino acids, with putative molecular weights ranging from 58.14 to 201.68 kDa and isoelectric points ranging from 5.20 to 9.24 (**Table 1**). Multiple alignments revealed amino acid frequencies at each position were conserved in the homologous domain sequence (**Figure 1B** and **Supplementary Figure S1**). For instance, all cotton *CRK* family members displayed three conserved cysteine residues in two stress-antifung domains (**Figure 1B**), which is a typical characteristic of the stress-antifung domain (C-X_8_-C-X_2_-C; X represents random residues) (Chen, 2001).

**Table 1:** Inventory and characteristics of the *CRK* genes identified in *Gossypium barbadense*.

Alias name	Gene ID	Chr	Chr. location	ORF length (bp)	Peptide length (aa)	MW (kDa)	pI	Number of introns
*GbCRK01*	GOBAR_AA34555	At_S0001	1337912–1343469	1836	611	67.32	5.91	6
*GbCRK02*	GOBAR_AA24536	At_S0006	70995864–71004670	2136	711	79.31	5.29	8
*GbCRK03*	GOBAR_AA24535	At_S0006	71005538–71008007	1953	650	72.10	5.20	6
*GbCRK04*	GOBAR_AA02193	At_S0006	99983841–99986324	2013	670	74.76	5.88	5
*GbCRK05*	GOBAR_AA02190	At_S0006	100001197–100004772	1887	628	70.64	9.24	6
*GbCRK06*	GOBAR_AA06767	At_S0006	115065828–115075528	5454	1817	201.68	6.15	9
*GbCRK07*	GOBAR_AA06768	At_S0006	115077555–115081009	1995	664	74.14	6.79	7
*GbCRK08*	GOBAR_AA03823	At_S0009	52655052–52657821	1917	638	70.53	6.30	6
*GbCRK09*	GOBAR_AA03826	At_S0009	52785439–52794757	1914	637	70.82	5.77	5
*GbCRK10*	GOBAR_AA25585	At_S0010	1364677–1369559	1986	661	74.09	5.68	5
*GbCRK11*	GOBAR_AA38379	At_S0010	1525918–1529193	1971	656	73.78	6.58	6
*GbCRK12*	GOBAR_AA02607	At_S0010	22853370–22856797	2043	680	75.44	6.73	6
*GbCRK13*	GOBAR_AA22880	At_S0011	91744–94415	1902	633	69.78	8.72	5
*GbCRK14*	GOBAR_AA38920	At_S0012	85245565–85249156	2406	801	88.88	6.79	9
*GbCRK15*	GOBAR_DD05003	Dt_S0001	1121081–1126929	2001	666	73.43	5.79	6
*GbCRK16*	GOBAR_DD26134	Dt_S0005	10634432–10637142	1929	642	70.95	6.49	7
*GbCRK17*	GOBAR_DD06001	Dt_S0005	39777117–39779680	1944	647	71.78	8.22	6
*GbCRK18*	GOBAR_DD09713	Dt_S0006	36871008–36873756	1959	652	72.63	6.89	6
*GbCRK19*	GOBAR_DD05620	Dt_S0006	37477588–37481568	2145	714	79.92	6.02	8
*GbCRK20*	GOBAR_DD03238	Dt_S0006	49266208–49268791	1950	649	72.33	8.27	6
*GbCRK21*	GOBAR_DD03241	Dt_S0006	49292282–49294109	1566	521	58.14	6.18	3
*GbCRK22*	GOBAR_DD17611	Dt_S0006	59606988–59616301	5451	1816	201.68	6.24	10
*GbCRK23*	GOBAR_DD21042	Dt_S0009	29020350–29023093	2043	680	76.17	8.19	6
*GbCRK24*	GOBAR_DD04095	Dt_S0009	29400668–29403738	2034	677	75.55	6.68	6
*GbCRK25*	GOBAR_DD04091	Dt_S0009	29557665–29559781	1575	524	58.28	6.71	5
*GbCRK26*	GOBAR_DD33839	Dt_S0010	1384433–1387690	1941	646	72.37	8.15	7
*GbCRK27*	GOBAR_DD36087	Dt_S0010	1420366–1425062	2019	672	75.14	6.51	5
*GbCRK28*	GOBAR_DD28832	Dt_S0010	12460012–12463446	2076	691	76.81	6.09	6
*GbCRK29*	GOBAR_DD28829	Dt_S0010	12496972–12499834	2013	670	75.97	8.10	7
*GbCRK30*	GOBAR_DD28469	Dt_S0012	41979461–41982186	1980	659	73.05	6.10	6

*Chr, Chromosome; pI, Isoelectronic point*.

### Structural and Phylogenetic Analyses of the Cotton *CRK* Gene Family

**FIGURE 2:**
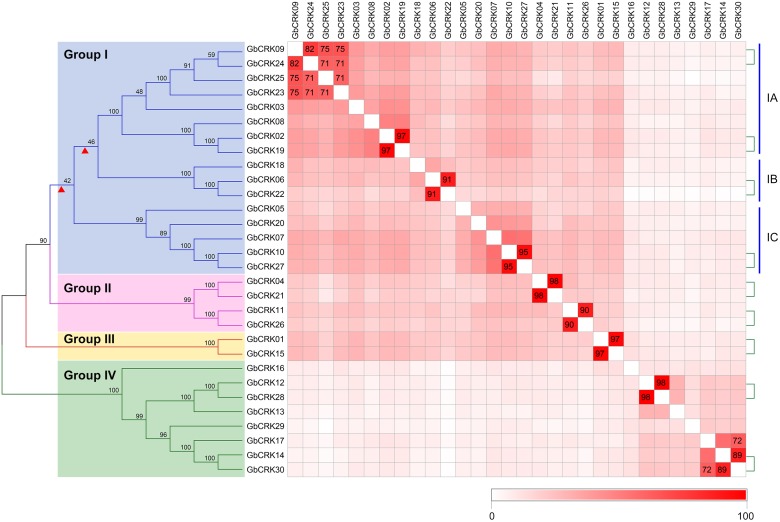
Phylogenetic analyses of *CRK* genes in *Gossypium barbadense*. Phylogenetic analyses divide the cotton *CRK* family into four subfamilies, here designated Group I–IV. Genes with similarities greater than 70% were plotted. The nine pairs of genes linked by the green-dotted lines are paralogous genes. Colored blocks from white to red represent the identity between genes from 0 to 100%; darker shades of red indicate higher levels of similarity.

Gene structure analysis of *GbCRKs* showed that the exon/intron organization varied in number and length (number of introns ranged from 3 to 10) (**Supplementary Figure S1**). Chromosome localization analysis revealed that 30 *GbCRKs* were distributed on 12 of the 26 chromosomes in cotton, and more than 30% of these genes (11 *GbCRKs*) were distributed on chromosomes A06 and D06 (**Supplementary Figure S2**). Similarity analysis revealed that 18 *GbCRKs* exhibited high identities (>80%), and that this similarity was observed pairwise, thus constituting nine different pairs (**Figure 2**), representative of nine paralogous pairs between the A and D sub-genome (**Supplementary Figure S2**). Chromosome physical location identification of the *GbCRKs* showed that most of them were present in tandem repeats (**Supplementary Table S2**); *GbCRK23*, *GbCRK24*, and *GbCRK25* were located in the same position on chromosome A09, whereas *GbCRK17* and *GbCRK30* which shared a high sequence identity (>70%) were located on different chromosomes (A05 and A07) (**Figure 2**), suggesting that tandem duplications and segmental duplications have occurred in cotton.

To assess sequence polymorphisms of the *CRK* gene family in cotton, an unrooted NJ tree was constructed using full-length amino-acid sequences. The cotton *CRK* gene family was classified into four subgroups (Group I–IV) with 2–16 members in each subgroup (**Figure 2**). Bootstrap values of each subgroup were high, especially for Group II, III, and IV (**Figure 2**), suggesting that the *GbCRKs* may be derived from a small set of genes and that duplication events and subsequent sequence evolution occurred during cotton genome evolution. As expected, nine paralogous pairs of *GbCRKs* in tandem or segmental duplications were clustered in the same branch due to their high identities (**Figure 2** and **Supplementary Figure S2**). The bootstrap values of 16 *GbCRKs* enriched in Group I were low among the different sub-clades (Group IA, IB, IC, ID) (**Figure 2**). Notably, all the *GbCRKs* in Group I are located on chromosomes 6 (A06 and D06) and 9 (A09 and D09) (11 and 5 CRKs, respectively) (**Figure 2** and **Supplementary Figure S2**).

Investigations of the gene structures in the nine pairs of paralogous *GbCRKs* also showed that the numbers or lengths of introns were variable, except for those of the paralogous pair, *GbCRK12* and *GbCRK28* (**Figure 2** and **Supplementary Figure S1**). Together, these results implied that the *CRK* gene family may have expanded by duplication from few common ancestral genes and followed by *CRK* sequence divergence in cotton.

### Comparative Analysis of the *CRK* Genes in Plant Genomes

**FIGURE 3:**
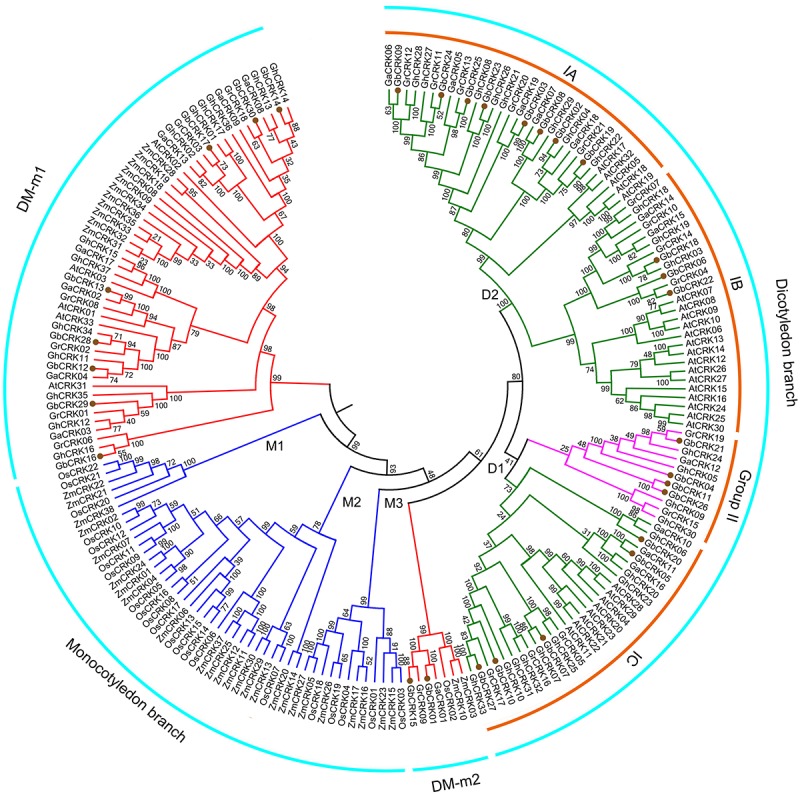
Phylogenetic relationships of *CRK* genes from seven plant species. An unrooted phylogenetic tree was constructed using the *CRK* nucleotide sequences from *G. barbadense*, *G. hirsutum*, *G. arboretum*, *G. raimondii*, *Arabidopsis thaliana*, *Oryza sativa*, and *Zea mays* using the NJ method with 1000 bootstrap replicates. Four groups are labeled as: dicotyledonous branch, DM-m1, DM-m2 and monocotyledonous branch. Subgroups are also labeled.

To gain insight into the evolution of the *CRK* gene family in cotton compared with other plant hosts, the phylogenetic relationships of CRK protein sequences from other six plant genomes were investigated by constructing an unrooted tree comprising representatives of three other cotton species, two monocotyledonous plants, *O. sativa* and *Z. mays*, and one dicotyledonous plant, *A. thaliana*. Using similar bioinformatics-driven methods, a total of 170 *CRK* genes were identified from these genome, including 21 in *G. raimondii* (*GrCRK01*–*GrCRK21*), 19 in *G. arboreum* (*GaCRK01*–*GaCRK19*), 37 in *G. hirsutum* (*GhCRK01*–*GaCRK37*), 33 in *A. thaliana* (*AtCRK01*–*AtCRK33*), 22 in *O. sativa* (*OsCRK01*–*OsCRK33*), and 38 in *Z. mays* (*ZmCRK01*–*ZmCRK33*) (**Supplementary Table S3**). Comparisons of the total number of *CRK* genes showed that the *CRK* gene family displayed expansion in the allotetraploid relative to the diploid cotton genome, but was not strictly associated with genome size, as the smallest genome, that of *A. thaliana*, encoded 33 *CRK* genes (**Supplementary Tables S2**, **S3**). The unrooted phylogenetic tree revealed that most of the *CRKs* were clustered according to dicot and monocot species. Three phylogenetic groups of CRK genes (M1–M3) clustered into monocot branches and two groups (D1 and D2) into dicot branches (**Figure 3**), respectively. The remaining *CRK* genes were clustered into two branches (DM-m1 and DM-m2), and contained genes from dicot and monocot species, while a small defined branch, DM-m2, contained *CRK* genes from all seven plant species (**Figure 3**). This finding suggested that dicot and monocot species shared the same set of ancient *CRK* genes, which later underwent diversification. Enrichment of *CRKs* in the phylogenetic branches was evident in branches shared by cotton and *A. thaliana* (**Figure 3**), suggesting that CRK duplication frequently occurs in dicots. Investigation of the *CRKs* from cotton showed that most of the *CRKs* can be clustered in small branches, concordant with the relationships between the four cotton species, and indicated that *CRK*s from *G. raimondii* and *G. arboreum* were donors to *G. barbadense* and *G. hirsutum* (**Figure 3**). Comparisons of unrooted trees showed that the *GbCRKs* were also clustered into four branches that correspond to the four groups of *GbCRKs* from *G. barbadense* (**Figures 2**, **3**). The *CRKs* in the small clade of the D2 branch originated from the cotton species corresponding to Group II in the *GbCRK* phylogenetic tree (**Figures 2**, **3**), forming a unique branch in cotton compared to *A. thaliana*, suggesting that these *CRKs* were specific to cotton. The *CRK* gene family displayed significant divergence between dicot and monocot plants, and tandem duplication of CRKs appears to be a major evolutionary mechanism in dicots.

### Expression Patterns of *GbCRK* Genes in Response to *Verticillium dahliae*

**FIGURE 4:**
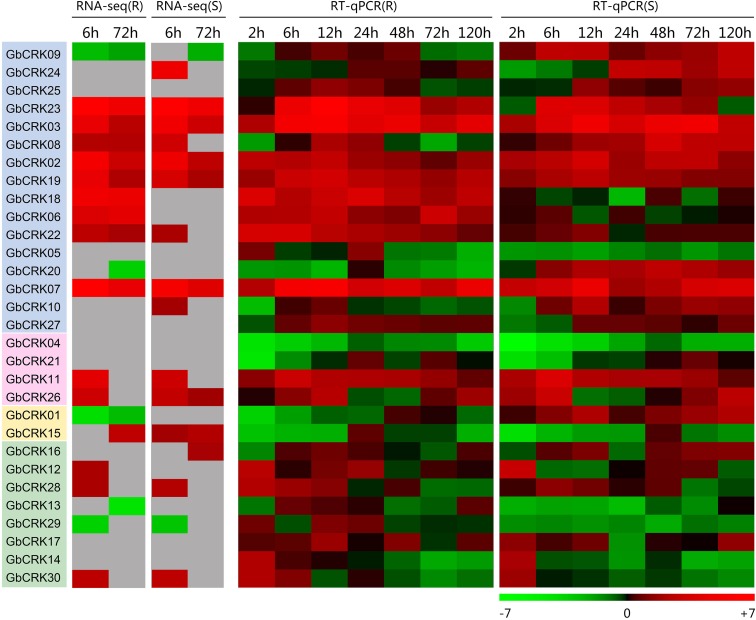
Expression patterns of *CRK* genes from Verticillium wilt resistant and susceptible *G. barbadense* cultivars. Both RNA-seq and RT-qPCR were used to examine the expression level of 30 *CRK* genes at different time points (6, 72 h) and (2, 6, 12, 24, 48, 72, and 120 h) after *V. dahliae* inoculation. A red box indicates up-regulation and green box indicates down-regulation. Color gradients indicate fold-change values (log_2_ values); with dark to light red representing increased transcript abundance and dark to light green indicating decreased transcript abundance. “R” indicates resistant *G. barbadense* cultivars and “S” indicates susceptible *G. barbadense* cultivars.

To gain insight into the role of the *GbCRK* genes in cotton disease resistance, the expression patterns of all 30 *GbCRK* genes were analyzed in response to *V. dahliae*. Two cultivars used in expression experiments were: *G. barbadense* cv. Hai 7124 and *G. barbadense* cv. GZ 57, which display resistance and susceptibility to VW, respectively. According to the reference RNA-seq data set of *G. barbadense* cv. Hai 7124 inoculated with *V. dahliae* (Chen et al., 2015), nine *GbCRK* genes were significantly up-regulated at the two evaluation points of 6 and 72 h after inoculation with *V. dahliae*, and all nine were clustered into Group I of the unrooted phylogenetic tree (**Figure 4**). RNA-seq analysis of the susceptible cotton cultivar *G. barbadense* cv. GZ 57 revealed that seven *GbCRK* genes were up-regulated at 6 and 72 h after inoculation with *V. dahliae* (**Figure 4**, unpublished RNA-seq data of the response of *G. barbadense* cv. GZ 57 to *V. dahliae* invasion). Interestingly, comparisons of the expression pattern of *GbCRKs* revealed that only four, *GbCRK06*, *GbCRK08*, *GbCRK18*, and *GbCRK22* (three of them belong to Group I), were up-regulated at both evaluation points in *G. barbadense* cv. Hai 7124 but were either down-regulated or unchanged in *G. barbadense* cv. GZ 57 following inoculation with *V. dahliae* (**Figure 4**), suggesting that these *GbCRK* genes played a role in cotton defense response to VW. RT-qPCR analyses confirmed *GbCRK* gene expression patterns, as these were consistent with RNA-seq results with the exception of *GbCRK08.* The RT-qPCR analyses of *GbCRK06*, *GbCRK18*, and *GbCRK22* that clustered in Group I revealed up-regulation of these genes at all evaluation points (2–120 h after inoculation with *V. dahliae*) in *G. barbadense* cv. Hai 7124 in response to *V. dahliae* (**Figure 4**). However, *GbCRK06*, *GbCRK18*, and *GbCRK22* were down-regulated or only slightly up-regulated at each comparable evaluation point in *G. barbadense* cv. GZ 57 in response to *V. dahliae* (**Figure 4**). These results suggested that the cotton *CRK* genes can be activated by *V. dahliae*, and that a subset of genes contributes to VW resistance in *G. barbadense*.

### Functional Characterization of *GbCRK* Genes Involved in Cotton Verticillium Wilt Resistance

**FIGURE 5:**
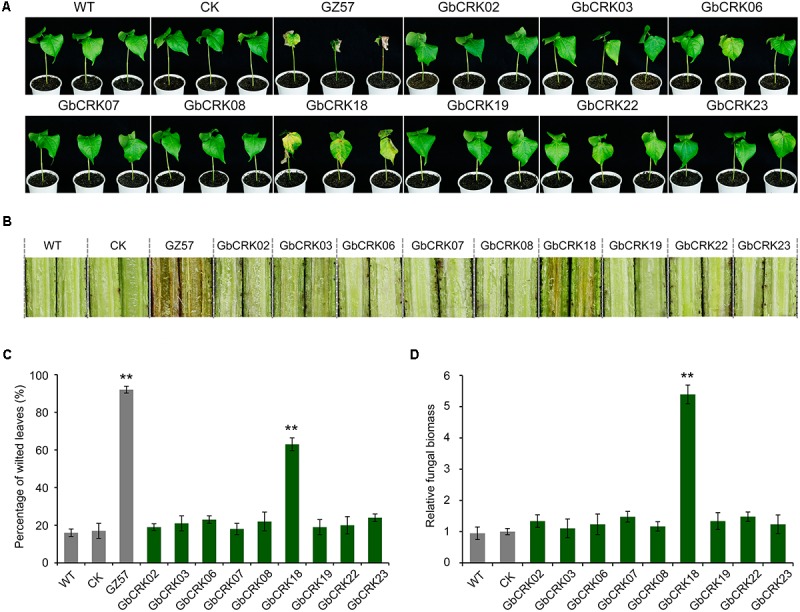
Virus-induced gene silencing of candidate *CRK*s implicated in VW resistance. **(A)** The VW phenotypes of wilting leaves in corresponding *CRK* gene-silenced plants post inoculation. Approximately 21 days after the VIGS procedure in the 2-week-old-resistant *G. barbadense* cv. Hai 7124, the pTRV2:00 (CK), the wild-type (WT), and the gene-silenced plants were inoculated with 20 mL of a 5 × 10^6^ conidia/mL suspension of *Verticillium dahliae* strain Vd991. Experiments consisted of three replicates of 12 plants each, and were arranged in a complete random block design. Error bars were calculated based on three biological replicates using standard deviation while relative gene quantifications were determined using a comparative threshold 2^-ΔΔCT^ method, ^∗∗^ indicates significant difference (*P* < 0.01). *GbCRK02*, *GbCRK03*, *GbCRK06*, *GbCRK07*, *GbCRK08*, *GbCRK18*, *GbCRK19*, *GbCRK22*, and *GbCRK23* represent gene-silenced plants and *G. barbadense* cv. GZ 57 was used as a negative control. **(B)** Stem vascular discoloration of CK, WT, and gene-silenced plants. Photographs were taken 4 weeks after inoculation with *V. dahliae* strain Vd991. **(C)** Percentage of diseased leaves after *V. dahliae* strain Vd991 inoculation. **(D)** Quantitative PCR analyses of fungal biomass of CK, WT, and gene-silenced plants. At least 12 inoculated plants and the Verticillium *EF-1α* gene were used as reference genes.

Expression patterns of *GbCRK* genes showed that nine *GbCRKs* in Group I were significantly up-regulated in cotton in response to *V. dahliae* infection (**Figure 4**). To identify the roles of these *GbCRKs* in VW resistance, tobacco rattle virus (TRV)-based VIGS was performed using the island cotton cv. Hai 7124 (VW-resistant cultivar). Fragments (∼500 bp) of all the nine *GbCRKs* amplified genes were separately integrated into the vector pTRV2 for generating gene-silenced cotton lines. Among the nine *GbCRK* genes in *G. barbadense*, the silencing of *GbCRK18* significantly impaired VW resistance in cv. Hai 7124; i.e., *GbCRK18*-silenced plants displayed severe symptoms of wilting leaves (**Figures 5A,C**), and displayed serious vascular discoloration 21 days after inoculation with *V. dahliae* Vd991 (**Figure 5B**). However, silencing of the eight other genes independently in cv. Hai 7124 did not change the resistance phenotypes 21 days after inoculation with *V. dahliae* Vd991 (**Figure 5**). Interestingly, the expression patterns of *GbCRK18* were sustainably up-regulated in resistant cotton, but down-regulated in the cotton susceptible to *V. dahliae* (**Figure 4**). Silencing *GbCRK06* and *GbCRK22* resulted in a similar expression pattern as that of *GbCRK18* in resistant and susceptible cotton, but did not compromise resistance to *V. dahliae* (**Figure 5A**). The RT-qPCR analyses indicated that the gene silencing efficiency of all nine *GbCRK* genes was greater than 70% (**Supplementary Figure S3**). Additionally, qPCR quantification of fungal biomass in cotton plants demonstrated that the function of *GbCRK18*, but not *GbCRK06* and *GbCRK22*, is associated with resistance, as there was increased fungal biomass associated with the *GbCRK18*-silenced plants that also displayed increased susceptibility to the pathogen (**Figure 5D**).

### Expression of *GbCRK18* in Response to *Verticillium dahliae* Is Modulated by Jasmonic Acid

**FIGURE 6:**
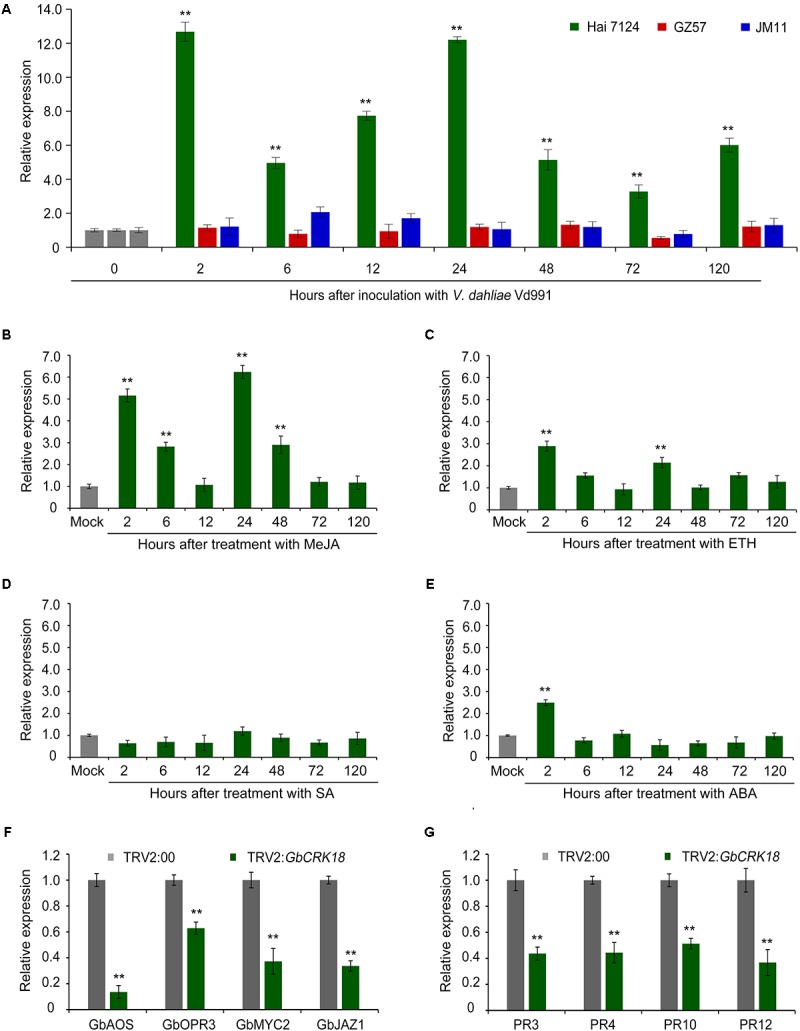
Expression pattern of *CRK18* under different treatments and of the defense marker genes in the *CRK18* transgenics. **(A)** Expression analysis of *GbCRK18* in cotton varieties Hai 7124, GZ 57, and JM 11 after inoculation with *V. dahliae* Vd991 by RT-qPCR. **(B**–**E)** Expression analysis of *GbCRK18* in *G. barbadense* cv. Hai 7124 in response to: **(B)** methyl jasmonic acid (MeJA), **(C)** ETH, **(D)** SA, and **(E)** ABA. Three-week-old *G. barbadense* plants were treated with 10 mM MeJA, 10 mM ETH, 10 mM SA, and 100 μM ABA, and harvested at 2, 6, 12, 24, 48, and 72 h, respectively. Relative expression analyses of *GbCRK18* by RT-qPCRs were performed using the cotton *18S* gene as a reference, and relative expression was compared with expression levels in cotton plants that were treated with sterile water (Mock). Transcript levels of genes related to **(F)** JA signaling pathways and **(G)** defense marker genes were analyzed in *GbCRK18*-silenced plants by RT-qPCR. Relative expression was calculated using the comparative threshold 2^-ΔΔCT^ method, and values represent averages of three independent biological replicates of three plants each. Error bars were calculated based on three biological replicates using standard deviation; ^∗∗^ indicates significant difference (*P* < 0.01).

The expression of *GbCRK18* was examined in the resistant *G. barbadense* cv. Hai 7124 after inoculation with the highly virulent *V. dahliae* strain Vd991, and quantified by RT-qPCR using the susceptible cotton lines *G. barbadense* cv. GZ 57 and *G. hirsutum* cv. JM 11 for comparison. Expression of *GbCRK18* was quickly and strongly up-regulated (*P* < 0.001) in the resistant variety cv. Hai 7124 at seven time points until 120 h, relative to the lower levels observed at the comparable time points for cv. GZ 57 and cv. JM11 (**Figure 6A**). *GbCRK18* was significantly up-regulated (*P* < 0.05) in cv. Hai 7124 after treatment with methyl jasmonic acid (MeJA) at several points within 120 h (**Figure 6B**), but was less affected by treatment with ethylene (ET) (**Figure 6C**) and not changed by treatment with SA or ABA (**Figures 6D,E**), which suggested that the *GbCRK18* response of the resistant cultivar to *V. dahliae* is mediated by jasmonic acid (JA) signaling. Interestingly, RT-qPCR analysis of MeJA-related genes between the *GbCRK18*-silenced line cv. Hai 7124 and control showed that four of the genes tested, including *GbAOS*, *GbOPR3*, *GbMYC2*, and *GbJAZ1*, were significantly down-regulated in *GbCRK18* silenced cotton that displayed susceptibility to *V. dahliae* (**Figure 6F**); moreover, the marker genes induced by MeJA including pathogenesis-related gene 3 (*PR3*), *PR4*, *PR10*, and *PR12*, were also significantly down-regulated in *GbCRK18*-silenced line cv. Hai 7124 after inoculation with *V. dahliae* (**Figure 6G**). These results suggested that *GbCRK18* mediates a VW defense response through JA signaling.

### GbCRK18 Is Localized to the Plasma Membrane

**FIGURE 7:**
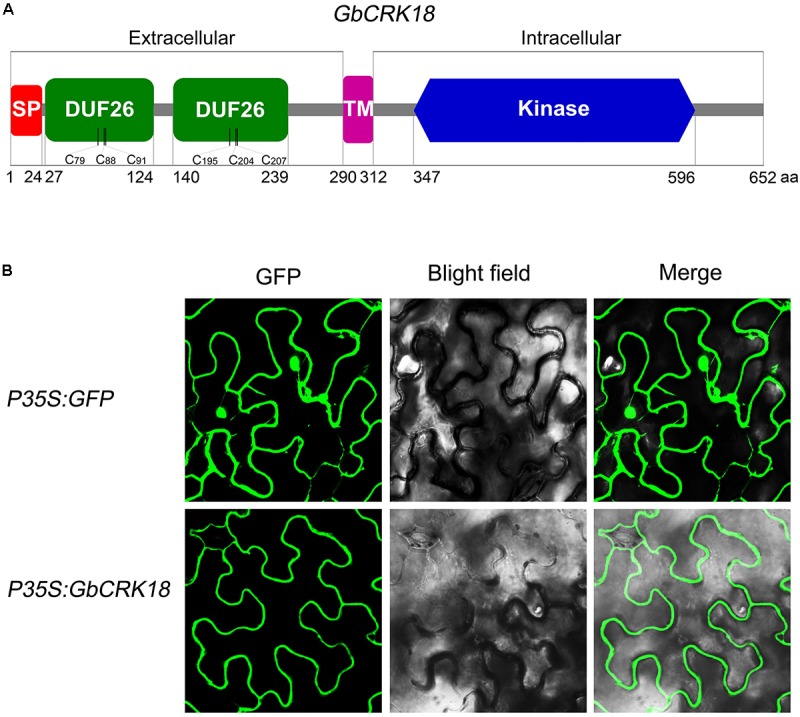
Protein structure model and subcellular localization of GbCRK18. **(A)** The conserved protein structure model of GbCRK18. Conserved domains of GbCRK18 including: a signal peptide (SP) from 1 to 24 amino acids; two stress-antifung domains (DUF26) from 27 to 124 and 140 to 239 amino acids; a transmembrane domain (TM) from 290 to 312 amino acids; and an intracellular kinase domain (Kinase) from 347 to 596 amino acids, respectively. **(B)** Subcellular localization of *GbCRK18.* For the subcellular localization of *GbCRK18*, a p35S:*GbCRK18* containing a full-length coding sequence of *GbCRK18* was inserted into the pBin-*GFP4* vector and introduced into tobacco by *Agrobacterium* infiltration. The p35S:*GFP* empty vector was used as a control and transformants were viewed with laser scanning confocal microscopy ×200 magnification with excitation and emission wavelengths of 488 and 510 nm, respectively.

Conserved domain analysis of the GbCRK18 peptide sequence using SMART identified a typical *CRK* family protein with a signal peptide and TM domain, which was confirmed by SignalP4.1 (1–24 aa,) and TMHMM2.0 (290–312 aa) (**Figure 7A**), respectively. To test this hypothesis, we assessed the subcellular location of GbCRK18 by transient expression in tobacco and compared this localization of the control *GFP* fusion. As predicted, the fusion protein was clearly localized to plasma membrane (**Figure 7B**), in contrast to the fluorescence signal of *GFP* proteins (p35S:*GFP*), which was prevalent throughout the foliar cells in tobacco (**Figure 7B**). An extracellular domain of stress-antifung domain (27–239 aa) and an intracellular domain of Pkinase (347–596 aa) were also identified, suggesting that *GbCRK18* is localized to the plasma membrane and may directly interact with pathogen-associated molecular patterns and mediate signal transduction via the intracellular domain.

## Discussion

Cotton (*Gossipium* spp.) is an important economic crop worldwide, and VW caused by *V. dahliae*, results in significant annual economic losses through reduced boll quality and quantity. Improving genetic resistance is the preferred method of managing VW in most crops (Schaible et al., 1951; Putt, 1964; Simko et al., 2004; Bolek et al., 2005; Zebrowska et al., 2006; Li et al., 2017), but identifying this resistance is difficult because of the unavailability of resistance genes against this pathogen. However, several genes that contribute to the defense response against VW have been reported in cotton, including *GbCAD1* and *GbSSI2* (Gao et al., 2013), *GbaNA1* (Li et al., 2018), *GbSTK* (Zhang Y. et al., 2013), *GbTLP1* (Munis et al., 2010), and *GbNRX1* (Li et al., 2016), etc. In spite of this, the *CRKs*, with known roles in plant resistance (Chen et al., 2003; Acharya et al., 2007; Ederli et al., 2011; Zhang X. et al., 2013; Yeh et al., 2015; Yadeta et al., 2017), have not been cloned from cotton and examined for roles in VW resistance. In this study, we systematically investigated the *CRK* genes found in a representative genome of *G. barbadense*, which contains abundant resistance gene resources among the different cotton species. These *CRK*s and their respective translated products were characterized based on structures, conserved domains, phylogenetic relationships among plants, and expression profiles in response to *V. dahliae.* Notably, we identified a candidate resistance gene, *GbCRK18* and its role in conferring VW resistance was confirmed by a gene silencing approach.

Plants that have been sequenced contain many copies of *RLKs*. For instance, over 600 and 1000 *RLKs* are contained in the genomes of *Arabidopsis* and rice, respectively (Shiu and Bleecker, 2003). *RLKs* have highly diverse extracellular regions, including one or more domains that are used to categorize RLKs into distinct subfamilies (Shiu and Bleecker, 2003). *CRKs* comprise a large subfamily of plant *RLKs* that possess an extracellular domain, a single-pass transmembrane domain, and an intracellular Ser/Thr protein kinase domain (Chen et al., 2003), important in disease resistance and apoptosis in plants (Chen et al., 2003; Acharya et al., 2007; Ederli et al., 2011). However, except for some well-studied examples in *Arabidopsis* (Chen et al., 2003; Acharya et al., 2007; Ederli et al., 2011; Zhang X. et al., 2013; Yeh et al., 2015; Yadeta et al., 2017), few *CRK* genes have been characterized for their role in plant disease resistance in cotton. Genomic analysis is an effective means of transferring knowledge from one taxon to another (Chen et al., 2003; Zhang Y. et al., 2012; Zhang et al., 2017; Xiao et al., 2017), allowing for a faster pace of gene discoveries associated with disease resistance.

In this study, we conducted genome-wide analyses of the *CRK* gene family in the four known genomes of cotton species, along with other three plant species; *O. Sativa*, *Z. mays*, and *A. thaliana* (**Supplementary Tables S2**, **S3**). Through data mining, *CRKs* were identified as transmembrane proteins characterized by the presence of two extracellular DUF26 domains (**Figure 1A**; Chen, 2001; Bourdais et al., 2015). Molecular phylogenetic analysis suggested a significantly divergent evolutionary history of *CRK* genes in dicot and monocot species, except for a common branch (DM-m1) enriched with the *CRKs* from sequenced plant genomes (**Figure 3**). *CRKs* from the same species tended to be clustered together in the same branch among dicot species but not monocot species, possibly implying that these *CRKs* originated from a few ancestral *CRK*s in dicot species and tandem or segmental duplication could have frequently occurred in dicot species relative to the monocot species.

The evolution of disease resistance genes is mediated by sequence exchange, tandem or segmental duplication events, or gene conversion (Bent et al., 1994; Hulbert et al., 2001; Leister, 2004). For instance, tandem and segmental duplications frequently occur in *NBS-LRR* gene clusters that have led to the formation of the phylogenetic lineage of *NBS-LRR* genes in the *Arabidopsis* genome (Baumgarten et al., 2003; Meyers et al., 2003), while sequence exchanges tend to homogenize the members of the Cf-9 gene cluster in tomato (Van der Hoorn et al., 2001). Among the 30 *GbCRKs* identified in *G. barbadense* in the current study, 14 and 16 genes were located in the A and D sub-genomes, respectively, while 18 other genes appeared to exist in orthologous pairs between the A and D sub-genomes (**Figure 2** and **Supplementary Figure S2**). We interpret this observation to mean that few segmental duplications may have occurred between different non-allelic chromosomes of *CRK* gene family in cotton. Our investigation of the physical location of *CRK* genes revealed that some of them are located closely and also showed high sequence identities such as *GbCRK23*, *GbCRK24*, and *GbCRK25* (**Supplementary Figure S2**), suggesting that tandem duplication is a main evolutionary mechanism of the *CRK* gene family in cotton. The total number of *CRK*s in the allotetraploid genomes were not more than twofold that of the diploid cotton genomes, and phylogenetic analysis also showed that most of the *CRK* genes had a corresponding ortholog in *G. arboretum* and *G. raimondii*, which are generally recognized as the donors of the allotetraploid cotton genome (**Figure 3**), suggesting that the *CRK* family expands through tandem duplication events.

Several *CRK* genes have been reported to play critical roles in disease resistance, including those from *Arabidopsis* (Acharya et al., 2007; Wrzaczek et al., 2010; Bourdais et al., 2015; Yeh et al., 2015; Yadeta et al., 2017), wheat (Yang et al., 2013), *Medicago truncatula* (Berrabah et al., 2014), barley (Rayapuram et al., 2012), and rice (Mawsheng et al., 2016). The functions of *CRKs* involved in disease resistance have been well studied in *Arabidopsis*, including *CRK4*, *CRK5*, *CRK6*, *CRK13*, *CRK19*, *CRK20*, *CRK28*, *CRK36*, and *CRK45* (Lee et al., 2017). For example, over-expression of *CRK5* and *CRK13* and *CRK28* in *Arabidopsis* led to enhanced resistance against *Pseudomonas syringae* pv. tomato (*Pst*) DC3000 (Chen et al., 2003; Acharya et al., 2007; Yadeta et al., 2017). However, involvement of *CRK* genes in VW resistance in cotton had not been reported except for the study by Zhang et al. (2018). In this study, we found that the members of the *CRK* gene family displayed up-regulation following infection by *V. dahliae* (**Figure 4**). One specific *CRK* gene (*GbCRK18*) participated in VW resistance and its silencing resulted in susceptibility to *V. dahliae* (**Figure 5**). Moreover, *CRKs* are also reported to participate in signal transduction of hormones such as ABA and abiotic stress (Zhu et al., 2007). In this study, we found that the expression of *GbCRK18* in response to JA (**Figure 6B**) follows a temporal expression pattern similar to the expression pattern observed following *V. dahliae* inoculation (**Figure 6A**); the expression of genes associated with JA biosynthesis (**Figure 6F**), suggested that JA-mediated signaling is important for VW resistance via *GbCRK18*. Plants infected by pathogens initiate a variety of defense responses by hormone signaling pathways, which lead to activation of defined sets of target genes (Feys and Parker, 2000). As expected, the expression of defense response marker genes in JA signaling (Mazarei et al., 2007) was also significantly reduced after silencing the *GbCRK18* in cotton (**Figure 6G**). The above results suggested that the *CRK* gene family, and particularly *GbCRK18*, plays an important role in VW resistance in cotton.

## Conclusion

Genes in the *CRK* family from *G. barbadense* genome were systematically analyzed. Silencing of *GbCRK18*, encoding a protein that is localized in the plasma membrane, compromised resistance against VW resistance in *G. barbadense*. Our study also demonstrated that the *CRK* gene family plays an important role in defense responses, and that *GbCRK18* is a functional gene that confers VW resistance in *G. barbadense*.

## Author Contributions

X-FD, J-YC, and KS conceived the study and designed all experiments. J-YC performed the data analysis and interpretation. T-GL wrote the paper and performed the gene family analysis. KS, DS, SK, ND, and AH revised the manuscript. D-DZ, LZ, and DW performed the gene silencing and gene expression analysis. Z-QK, J-JL, B-LW, and C-MY performed the pathogenicity analysis.

## Conflict of Interest Statement

The authors declare that the research was conducted in the absence of any commercial or financial relationships that could be construed as a potential conflict of interest.

## Footnotes

1 http://pfam.xfam.org/

2 http://meme-suite.org/

3 http://www.softsea.com/review/MapInspect.html

4 http://www.megasoftware.net

5 http://gsds1.cbi.pku.edu.cn/
