# Supplementary material for: Genome-Wide Identification and Functional Analyses of the CRK Gene Family in Cotton Reveals GbCRK18 Confers Verticillium Wilt Resistance in Gossypium barbadense
Source: Front Plant Sci. 2018 Sep 11;9:1266. doi: 10.3389/fpls.2018.01266 (PMC6141769; doi:10.3389/fpls.2018.01266)
Supplement: Supplementary file 1 [file Table_1.PDF]

Table S1 | Information of the PCR primers used in this study.

Primer name	Primer sequence (5′ -3′)	Purpose
**Primers for expression analysis of *Gossypium barbadense* CRK family**		
GbCRK01 -F	TTGCTGGTGATACTCGTAG	Primers used for qRT-PCR of *GbCRK01*
GbCRK01 -R	TCTCGTAATCGGCTTGTG	Primers used for qRT-PCR of *GbCRK01*
GbCRK02 -F	GTATCAGCACAGCAGGTAA	Primers used for qRT-PCR of *GbCRK02*
GbCRK02 -R	CCAGCAGACAGGTTATAGAA	Primers used for qRT-PCR of *GbCRK02*
GbCRK03 -F	CTCTTCATTCTCCTCAATCAC	Primers used for qRT-PCR of *GbCRK03*
GbCRK03 -R	TGGCTGCTTCCTTCCTAT	Primers used for qRT-PCR of *GbCRK03*
GbCRK04 -F	TTCATATCGGACTGTTGTGT	Primers used for qRT-PCR of *GbCRK04*
GbCRK04 -R	GTTGTTGTGGTTGGCTTAG	Primers used for qRT-PCR of *GbCRK04*
GbCRK05 -F	CAGCAGTTCCTCGGTTAC	Primers used for qRT-PCR of *GbCRK05*
GbCRK05 -R	AGGCACTACTTGTTGATTGA	Primers used for qRT-PCR of *GbCRK05*
GbCRK06 -F	GGTCCAACTCCTCAATCTG	Primers used for qRT-PCR of *GbCRK06*
GbCRK06 -R	ATTCTTCCTCCGCTTCATC	Primers used for qRT-PCR of *GbCRK06*
GbCRK07 -F	AGTTCCACCACCTCCATT	Primers used for qRT-PCR of *GbCRK07*
GbCRK07 -R	CTTCCTCCTTGCTCTCCT	Primers used for qRT-PCR of *GbCRK07*
GbCRK08 -F	GCTGCTATGGAAGGATGG	Primers used for qRT-PCR of *GbCRK08*
GbCRK08 -R	CGGAGAAGAAGTTGTTGGA	Primers used for qRT-PCR of *GbCRK08*
GbCRK09 -F	TTGGTATGATATGGCAGTGT	Primers used for qRT-PCR of *GbCRK09*
GbCRK09 -R	TTGGTGAAGGTAGAGAAGTC	Primers used for qRT-PCR of *GbCRK09*
GbCRK10 -F	GGACAGTTGGATTGGTTGA	Primers used for qRT-PCR of *GbCRK10*
GbCRK10 -R	ACGGTGGATGATTCTAAGC	Primers used for qRT-PCR of *GbCRK10*
GbCRK11 -F	CAAGACCTTAGAAGCAATCG	Primers used for qRT-PCR of *GbCRK11*
GbCRK11 -R	CATCAACTCGGAATCATACG	Primers used for qRT-PCR of *GbCRK11*
GbCRK12 -F	ATCAATGGCAGAAGTGGTT	Primers used for qRT-PCR of *GbCRK12*
GbCRK12 -R	GGTAGTGTCGGAGGAAGT	Primers used for qRT-PCR of *GbCRK12*
GbCRK13 -F	GTGCTTGGTGCTATTGGT	Primers used for qRT-PCR of *GbCRK13*
GbCRK13 -R	TGCTCCTCCTTGTCCTAAT	Primers used for qRT-PCR of *GbCRK13*
GbCRK14 -F	TGCGGTTCTTCCTCAATG	Primers used for qRT-PCR of *GbCRK14*
GbCRK14 -R	GCTATAATTCTCGGCTCTCA	Primers used for qRT-PCR of *GbCRK14*
GbCRK15 -F	GCTTGGTTACATTGGAACAA	Primers used for qRT-PCR of *GbCRK15*
GbCRK15 -R	GTTACGGAGTGACTGCTAA	Primers used for qRT-PCR of *GbCRK15*
GbCRK16 -F	AATCTGGTTCGCCTTGTT	Primers used for qRT-PCR of *GbCRK16*
GbCRK16 -R	TGCTGTGCCTACTATTATGT	Primers used for qRT-PCR of *GbCRK16*
GbCRK17 -F	CCTGATGCTAACTATGGTCTA	Primers used for qRT-PCR of *GbCRK17*
GbCRK17 -R	TGTCTTGCTGATGCTTGAA	Primers used for qRT-PCR of *GbCRK17*
GbCRK18 -F	AAGCAAGGAGGTTATGTTCA	Primers used for qRT-PCR of *GbCRK18*
GbCRK18 -R	AAGGATGGAGCAGTAGTAGA	Primers used for qRT-PCR of *GbCRK18*
GbCRK19 -F	GTATCAGCACAGCAGGTAA	Primers used for qRT-PCR of *GbCRK19*
GbCRK19 -R	CAGGTTATAGAAGCCATAGTTG	Primers used for qRT-PCR of *GbCRK19*
GbCRK20 -F	CGTGCTGCGTATGATGAT	Primers used for qRT-PCR of *GbCRK20*
GbCRK20 -R	TAGGTGAGGAAGGCGAAG	Primers used for qRT-PCR of *GbCRK20*
GbCRK21 -F	TTCTGTTCGGTCGTAATGAT	Primers used for qRT-PCR of *GbCRK21*
GbCRK21 -R	ATGGCAATCGTCGTATGAA	Primers used for qRT-PCR of *GbCRK21*
GbCRK22 -F	GGTCCAACTCCTCAATCTG	Primers used for qRT-PCR of *GbCRK22*
GbCRK22 -R	ATTCTTCCTCCGCTTCATC	Primers used for qRT-PCR of *GbCRK22*
GbCRK23 -F	GGCTTCTCTACCTTCATCAA	Primers used for qRT-PCR of *GbCRK23*
GbCRK23 -R	CATCTCTGCGTCCAACAA	Primers used for qRT-PCR of *GbCRK23*
GbCRK24 -F	CAAGAAGTGAGGTGATGAGA	Primers used for qRT-PCR of *GbCRK24*
GbCRK24 -R	CCAGCAAGCATTAGAACAAT	Primers used for qRT-PCR of *GbCRK24*
GbCRK25 -F	CCAATCTTCAGCACAGGAA	Primers used for qRT-PCR of *GbCRK25*
GbCRK25 -R	ATTAGCACAACCGCACTC	Primers used for qRT-PCR of *GbCRK25*
GbCRK26 -F	GATTATCGCAGTCGTATGTG	Primers used for qRT-PCR of *GbCRK26*
GbCRK26 -R	ATTCGCCTTCTTGTGTTATG	Primers used for qRT-PCR of *GbCRK26*

GbCRK27 -F	CCTTCTCCAACACCTTCTC	Primers used for qRT-PCR of *GbCRK27*
GbCRK27 -R	TAACAGCCAACACATCAGTA	Primers used for qRT-PCR of *GbCRK27*
GbCRK28 -F	ATCAATGGCAGAAGTGGTT	Primers used for qRT-PCR of *GbCRK28*
GbCRK28 -R	GGTAGTGTCGGAGGAAGT	Primers used for qRT-PCR of *GbCRK28*
GbCRK29 -F	CCAGGATGGTTCACAAGG	Primers used for qRT-PCR of *GbCRK29*
GbCRK29 -R	GAGACAAGAGGAGCACATT	Primers used for qRT-PCR of *GbCRK29*
GbCRK30 -F	ACGGTTCTTCCTCAATGC	Primers used for qRT-PCR of *GbCRK30*
GbCRK30 -R	GCTATAATTCTCGGCTCTCA	Primers used for qRT-PCR of *GbCRK30*
**VIGS fragment amplification of nine genes**		
VIGS-GbCRK02-F	GTATCAGCACAGCAGGTAA	Amplification of VIGS fragment of *GbCRK02*
VIGS-*GbCRK02* -R	GACAAGAATCAGGAGAGGTT	Amplification of VIGS fragment of *GbCRK02*
VIGS-*GbCRK03* -F	AACCTCAACAACCTCCTCT	Amplification of VIGS fragment of *GbCRK03*
VIGS-*GbCRK03* -R	GTCCGCATCCGTAACATT	Amplification of VIGS fragment of *GbCRK03*
VIGS-*GbCRK06* -F	AACCTACTCCTCACTTCTCT	Amplification of VIGS fragment of *GbCRK06*
VIGS-*GbCRK06* -R	CCTCAGCCATACCAGTTG	Amplification of VIGS fragment of *GbCRK06*
VIGS-*GbCRK07* -F	CTTGACTTATACGACCACTTG	Amplification of VIGS fragment of *GbCRK07*
VIGS-*GbCRK07* -R	TACCACCTCCAGAGTTGAA	Amplification of VIGS fragment of *GbCRK07*
VIGS-*GbCRK08* -F	ACACTTGCCTCAACTCTAC	Amplification of VIGS fragment of *GbCRK08*
VIGS-*GbCRK08* -R	TGGTCTAAGAATCCTACATCC	Amplification of VIGS fragment of *GbCRK08*
VIGS-*GbCRK18* -F	TTCTACATTCCACGAACTCA	Amplification of VIGS fragment of *GbCRK18*
VIGS-*GbCRK18* -R	ACATAACCTCCTTGCTTCC	Amplification of VIGS fragment of *GbCRK18*
VIGS-*GbCRK19* -F	GTATCAGCACAGCAGGTAA	Amplification of VIGS fragment of *GbCRK19*
VIGS-*GbCRK19* -R	GACAAGAATCAGGAGAGGTT	Amplification of VIGS fragment of *GbCRK19*
VIGS-*GbCRK22* -F	GGCTGTCCATCTGTTATTCA	Amplification of VIGS fragment of *GbCRK22*
VIGS-*GbCRK22* -R	GCATCGGTATCTTCATCGT	Amplification of VIGS fragment of *GbCRK22*
VIGS-*GbCRK23* -F	ACCAGCAACAGCACTTAC	Amplification of VIGS fragment of *GbCRK23*
VIGS-*GbCRK23-* R	CACTTGAACGACATCTCTTAG	Amplification of VIGS fragment of *GbCRK23*
**Expression detection of the key genes in JA pathway**		
GbAOS -F	TGCCACCTGGTCCTTTCATTTC	qRT-PCR for *GbAOS* gene
GbAOS -R	GCGTGTTTGGGCTCGGAAGGGTCG	qRT-PCR for *GbAOS* gene
GbOPR3 -F	AGAGGTCCACTCCTGGCGGCTT	qRT-PCR for *GbOPR3* gene
GbOPR3 -R	CCACCTGTTCTTCATTGTAGATTCC	qRT-PCR for *GbOPR3* gene
GbMYC2 -F	GCTCCGCCACTACCGTGCTC	qRT-PCR for *GbMYC2* gene
GbMYC2 -R	CTCGAAGCACTTTTTTACGGTGTTC	qRT-PCR for *GbMYC2* gene
GbJAZ1 -F	AGCCTCAAAAAGGAAGACCTCAAAC	qRT-PCR for *GbJAZ1* gene
GbJAZ1 -R	TGGCTGCTCAATCACCATAGTAATC	qRT-PCR for *GbJAZ1* gene
**Expression detection of resistance marker genes ralated to JA pathway**		
PR3 -F	TGGTGTTGGCAGCATTATCAC	qRT-PCR for *PR3* gene
PR3 -R	CTCCGGTTGTGCCAAATCCA	qRT-PCR for *PR3* gene
PR4 -F	GAGGGTAAGAAACTCAAGGACTGG	qRT-PCR for *PR4* gene
PR4 -R	CTCCATCAGTGTCCAATCGGTT	qRT-PCR for *PR4* gene
PR10 -F	GAGTTATGAGTTTGAGGTAA	qRT-PCR for *PR10* gene
PR10 -R	GGACTAGCATCACCTTCGAG	qRT-PCR for *PR10* gene
PR12 -F	TTTGCTGCTTTCGACGCAC	qRT-PCR for *PR12* gene
PR12 -R	CGCAAACCCCTGACCATG	qRT-PCR for *PR12* gene
**Primers for fungal biomass detection**		
Gh-18S -F	CGGCTACCACATCCAAGGAA	Reference for qRT-PCR
Gh-18S -R	TGTCACTACCTCCCCGTGTCA	Reference for qRT-PCR
Vd-EF-1α -F	TGAGTTCGAGGCTGGTATCT	Primers used for quantative PCR of *Verticillium dahliae*
Vd-EF-1α -R	CACTTGGTGGTGTCCATCTT	Primers used for quantative PCR of *Verticillium dahliae*
