# Supplementary material for: Genome-Wide Identification and Functional Analyses of the CRK Gene Family in Cotton Reveals GbCRK18 Confers Verticillium Wilt Resistance in Gossypium barbadense
Source: Front Plant Sci. 2018 Sep 11;9:1266. doi: 10.3389/fpls.2018.01266 (PMC6141769; doi:10.3389/fpls.2018.01266)
Supplement: Supplementary file 2 [file Table_2.PDF]

Table S2 | Information of all candidate CRK family genes in *Gossypium barbadense*.

Chromosome number	Starting position	Termination position	Candidate gene ID	Classical CRK gene	Alias name	Signal peptide domain	DUF26-1 domain	DUF26-2 domain	Transmembrane domain	Kinase domain	EggNOG domain annotation
At_S0001	1337834	1343496	gnl|CHGC|GOBAR_AA34555	Yes	*GbCRK01*	1	1	1	1	1	Cysteine-rich receptor-like protein kinase
At_S0005	52796136	52799846	gnl|CHGC|GOBAR_AA00191	No		0	1	1	1	1	Cysteine-rich receptor-like protein kinase
At_S0005	52814154	52819833	gnl|CHGC|GOBAR_AA00192	No		1	1	1	0	1	Cysteine-rich receptor-like protein kinase
At_S0006	100017411	100019952	gnl|CHGC|GOBAR_AA02188	No		0	1	1	1	1	Cysteine-rich receptor-like protein kinase
At_S0006	100007440	100010029	gnl|CHGC|GOBAR_AA02189	No		0	1	0	1	1	Cysteine-rich receptor-like protein kinase
At_S0006	100000944	100005062	gnl|CHGC|GOBAR_AA02190	Yes	*GbCRK02*	1	1	1	1	1	Cysteine-rich receptor-like protein kinase
At_S0006	99983841	99986324	gnl|CHGC|GOBAR_AA02193	Yes	*GbCRK03*	1	1	1	1	1	Pfam:DUF26
At_S0006	115065601	115075604	gnl|CHGC|GOBAR_AA06767	Yes	*GbCRK04*	1	1	1	1	1	Cysteine-rich receptor-like protein kinase
At_S0006	115077358	115081021	gnl|CHGC|GOBAR_AA06768	Yes	*GbCRK05*	1	1	1	1	1	Cysteine-rich receptor-like protein kinase
At_S0006	71005538	71008007	gnl|CHGC|GOBAR_AA24535	Yes	*GbCRK06*	1	1	1	1	1	Cysteine-rich receptor-like protein kinase
At_S0006	70995795	71004862	gnl|CHGC|GOBAR_AA24536	Yes	*GbCRK07*	1	1	1	1	1	Cysteine-rich receptor-like protein kinase
At_S0006	70881611	70885606	gnl|CHGC|GOBAR_AA24539	No		1	1	1	0	1	Cysteine-rich receptor-like protein kinase
At_S0006	102218441	102221363	gnl|CHGC|GOBAR_AA30807	No		1	1	1	0	1	Cysteine-rich receptor-like protein kinase
At_S0007	91713345	91716018	gnl|CHGC|GOBAR_AA00491	No		0	1	1	1	1	Cysteine-rich receptor-like protein kinase
At_S0009	52654832	52708358	gnl|CHGC|GOBAR_AA03823	Yes	*GbCRK08*	1	1	1	1	1	Cysteine-rich receptor-like protein kinase
At_S0009	52785437	52794759	gnl|CHGC|GOBAR_AA03826	Yes	*GbCRK09*	1	1	1	1	1	Cysteine-rich receptor-like protein kinase
At_S0009	49556462	49561848	gnl|CHGC|GOBAR_AA13838	No		0	1	1	0	1	Cysteine-rich receptor-like protein kinase
At_S0009	69369685	69371958	gnl|CHGC|GOBAR_AA19158	No		0	1	1	0	1	Pfam:DUF26
At_S0010	22851565	22857120	gnl|CHGC|GOBAR_AA02607	Yes	*GbCRK10*	1	1	1	1	1	Cysteine-rich receptor-like protein kinase
At_S0010	22894082	22897001	gnl|CHGC|GOBAR_AA02609	No		0	1	1	1	1	Cysteine-rich receptor-like protein kinase
At_S0010	1364552	1369802	gnl|CHGC|GOBAR_AA25585	Yes	*GbCRK11*	1	1	1	1	1	Cysteine-rich receptor-like protein kinase
At_S0010	1525740	1529464	gnl|CHGC|GOBAR_AA38379	Yes	*GbCRK12*	1	1	1	1	1	Pfam:DUF26
At_S0010	1541950	1547294	gnl|CHGC|GOBAR_AA38380	No		1	1	0	0	1	Cysteine-rich receptor-like protein kinase
At_S0011	91744	94626	gnl|CHGC|GOBAR_AA22880	Yes	*GbCRK13*	1	1	1	1	1	Pfam:DUF26
At_S0012	93555070	93560184	gnl|CHGC|GOBAR_AA11363	No		0	1	1	1	1	Cysteine-rich receptor-like protein kinase
At_S0012	85284269	85287139	gnl|CHGC|GOBAR_AA35362	No		0	1	1	1	1	Cysteine-rich receptor-like protein kinase
At_S0012	85245565	85249156	gnl|CHGC|GOBAR_AA38920	Yes	*GbCRK14*	1	1	1	1	1	Cysteine-rich receptor-like protein kinase
Dt_S0001	1121079	1126931	gnl|CHGC|GOBAR_DD05003	Yes	*GbCRK15*	1	1	1	1	1	Cysteine-rich receptor-like protein kinase
Dt_S0005	39776947	39781700	gnl|CHGC|GOBAR_DD06001	Yes	*GbCRK16*	1	1	1	1	1	Cysteine-rich receptor-like protein kinase
Dt_S0005	39966322	39968825	gnl|CHGC|GOBAR_DD06004	No		0	1	0	1	1	Cysteine-rich receptor-like protein kinase
Dt_S0005	15480442	15483432	gnl|CHGC|GOBAR_DD16157	No		1	1	1	0	1	Cysteine-rich receptor-like protein kinase
Dt_S0005	10631740	10637863	gnl|CHGC|GOBAR_DD26134	Yes	*GbCRK17*	1	1	1	1	1	Cysteine-rich receptor-like protein kinase
Dt_S0006	49266206	49268793	gnl|CHGC|GOBAR_DD03238	Yes	*GbCRK18*	1	1	1	1	1	Cysteine-rich receptor-like protein kinase
Dt_S0006	49292282	49294109	gnl|CHGC|GOBAR_DD03241	Yes	*GbCRK19*	1	1	1	1	1	Pfam:DUF26
Dt_S0006	37477514	37481962	gnl|CHGC|GOBAR_DD05620	Yes	*GbCRK20*	1	1	1	1	1	Cysteine-rich receptor-like protein kinase
Dt_S0006	37551049	37554208	gnl|CHGC|GOBAR_DD05621	No		1	1	1	0	1	Cysteine-rich receptor-like protein kinase
Dt_S0006	36852639	36858040	gnl|CHGC|GOBAR_DD09712	No		0	1	1	0	1	Cysteine-rich receptor-like protein kinase
Dt_S0006	36871008	36873845	gnl|CHGC|GOBAR_DD09713	Yes	*GbCRK21*	1	1	1	1	1	Cysteine-rich receptor-like protein kinase
Dt_S0006	57374844	57391021	gnl|CHGC|GOBAR_DD12466	No		0	1	1	0	1	Cysteine-rich receptor-like protein kinase
Dt_S0006	57400599	57408726	gnl|CHGC|GOBAR_DD12467	No		1	1	0	1	1	Cysteine-rich receptor-like protein kinase
Dt_S0006	59618355	59636589	gnl|CHGC|GOBAR_DD17609	No		0	1	0	1	1	Cysteine-rich receptor-like protein kinase
Dt_S0006	59618355	59626548	gnl|CHGC|GOBAR_DD17610	No		0	1	0	1	1	Cysteine-rich receptor-like protein kinase
Dt_S0006	59606777	59616376	gnl|CHGC|GOBAR_DD17611	Yes	*GbCRK22*	1	1	1	1	1	Cysteine-rich receptor-like protein kinase
Dt_S0006	49115520	49118749	gnl|CHGC|GOBAR_DD28041	No		0	1	0	0	1	Cysteine-rich receptor-like protein kinase
Dt_S0006	24211989	24215879	gnl|CHGC|GOBAR_DD37580	No		0	1	0	0	1	Cysteine-rich receptor-like protein kinase
Dt_S0006	46214563	46217304	gnl|CHGC|GOBAR_DD37638	No		1	1	1	0	1	Cysteine-rich receptor-like protein kinase
Dt_S0007	53631264	53634242	gnl|CHGC|GOBAR_DD05732	No		0	1	1	1	1	Cysteine-rich receptor-like protein kinase
Dt_S0008	4240661	4246152	gnl|CHGC|GOBAR_DD26311	No		1	1	1	0	1	Cysteine-rich receptor-like protein kinase
Dt_S0009	29557663	29559783	gnl|CHGC|GOBAR_DD04091	Yes	*GbCRK23*	1	1	1	1	1	Cysteine-rich receptor-like protein kinase
Dt_S0009	29493883	29503478	gnl|CHGC|GOBAR_DD04094	No		0	1	1	1	1	Cysteine-rich receptor-like protein kinase
Dt_S0009	29400668	29403738	gnl|CHGC|GOBAR_DD04095	Yes	*GbCRK24*	1	1	1	1	1	Cysteine-rich receptor-like protein kinase
Dt_S0009	29386820	29392252	gnl|CHGC|GOBAR_DD04096	No		0	1	1	1	1	Cysteine-rich receptor-like protein kinase
Dt_S0009	29020350	29023727	gnl|CHGC|GOBAR_DD21042	Yes	*GbCRK25*	1	1	1	1	1	Cysteine-rich receptor-like protein kinase
Dt_S0009	41668806	41671080	gnl|CHGC|GOBAR_DD23434	No		0	1	1	0	1	Pfam:DUF26
Dt_S0010	1767071	1771684	gnl|CHGC|GOBAR_DD10781	No		0	1	1	1	1	Cysteine-rich receptor-like protein kinase
Dt_S0010	12496970	12499836	gnl|CHGC|GOBAR_DD28829	Yes	*GbCRK26*	1	1	1	1	1	Cysteine-rich receptor-like protein kinase
Dt_S0010	12459394	12463806	gnl|CHGC|GOBAR_DD28832	Yes	*GbCRK27*	1	1	1	1	1	Cysteine-rich receptor-like protein kinase
Dt_S0010	1384329	1387938	gnl|CHGC|GOBAR_DD33839	Yes	*GbCRK28*	1	1	1	1	1	Pfam:DUF26
Dt_S0010	1392574	1397361	gnl|CHGC|GOBAR_DD33840	No		1	1	1	0	1	Cysteine-rich receptor-like protein kinase
Dt_S0010	1412454	1415712	gnl|CHGC|GOBAR_DD36086	No		0	1	0	1	1	Cysteine-rich receptor-like protein kinase
Dt_S0010	1420251	1425282	gnl|CHGC|GOBAR_DD36087	Yes	*GbCRK29*	1	1	1	1	1	Cysteine-rich receptor-like protein kinase
Dt_S0012	41978092	41982863	gnl|CHGC|GOBAR_DD28469	Yes	*GbCRK30*	1	1	1	1	1	Cysteine-rich receptor-like protein kinase
Dt_S0012	41971516	41974951	gnl|CHGC|GOBAR_DD28470	No		0	1	1	1	1	Cysteine-rich receptor-like protein kinase
