# Supplementary material for: Genome-Wide Identification and Functional Analyses of the CRK Gene Family in Cotton Reveals GbCRK18 Confers Verticillium Wilt Resistance in Gossypium barbadense
Source: Front Plant Sci. 2018 Sep 11;9:1266. doi: 10.3389/fpls.2018.01266 (PMC6141769; doi:10.3389/fpls.2018.01266)
Supplement: Supplementary file 3 [file Table_3.PDF]

Table S3 | Information of all CRK family genes in the remaining six species.

Candidate gene ID	Classical CRK gene	Alias name	Signal peptide domain	DUF26-1 domain	DUF26-2 domain	Transmembrane domain	Kinase domain
Gossypium hirsutum							
Gh_A05G1430	No		0	1	1	0	1
Gh_A05G2720	Yes	*GhCRK01*	1	1	1	1	1
Gh_A05G2721	Yes	*GhCRK02*	1	1	1	1	1
Gh_A06G1065	No		0	1	0	1	1
Gh_A06G1066	No		1	1	0	1	1
Gh_A06G1067	Yes	*GhCRK03*	1	1	1	1	1
Gh_A06G1068	No		0	1	1	0	1
Gh_A06G1073	Yes	*GhCRK04*	1	1	1	1	1
Gh_A06G1074	No		1	1	0	0	1
Gh_A06G1265	No		1	1	1	0	1
Gh_A06G1268	Yes	*GhCRK05*	1	1	1	1	1
Gh_A06G1270	No		1	1	1	0	1
Gh_A06G1271	Yes	*GhCRK06*	1	1	1	1	1
Gh_A06G1272	No		0	1	1	1	1
Gh_A06G1783	Yes	*GhCRK07*	1	1	1	1	1
Gh_A06G1784	No		1	1	1	0	1
Gh_A07G2037	No		0	1	1	1	1
Gh_A09G0665	Yes	*GhCRK08*	1	1	1	1	1
Gh_A09G0671	No		0	1	1	1	1
Gh_A09G0672	No		1	1	1	0	1
Gh_A09G1550	No		0	1	1	1	1
Gh_A10G0156	No		0	1	1	1	1
Gh_A10G0157	Yes	*GhCRK09*	1	1	1	1	1
Gh_A10G0159	No		1	1	0	0	1
Gh_A10G0160	No		0	1	1	1	1
Gh_A10G0161	Yes	*GhCRK10*	1	1	1	1	1
Gh_A10G0923	Yes	*GhCRK11*	1	1	1	1	1
Gh_A10G0924	Yes	*GhCRK12*	1	1	1	1	1
Gh_A10G0989	No		0	1	0	0	1
Gh_A11G0008	No		1	1	0	1	1
Gh_A12G1328	Yes	*GhCRK13*	1	1	1	1	1
Gh_A12G1329	Yes	*GhCRK14*	1	1	1	1	1
Gh_A12G1330	Yes	*GhCRK15*	1	1	1	1	1
Gh_D05G1086	Yes	*GhCRK16*	1	1	1	1	1

Candidate gene ID	Classical CRK gene	Alias name	Signal peptide domain	DUF26-1 domain	DUF26-2 domain	Transmembrane domain	Kinase domain
Gh_D05G1603	No		0	1	1	0	1
Gh_D05G3023	Yes	*GhCRK17*	1	1	1	1	1
Gh_D06G1293	Yes	*GhCRK18*	1	1	1	1	1
Gh_D06G1294	Yes	*GhCRK19*	1	1	1	1	1
Gh_D06G1295	Yes	*GhCRK20*	1	1	1	1	1
Gh_D06G1301	Yes	*GhCRK21*	1	1	1	1	1
Gh_D06G1302	Yes	*GhCRK22*	1	1	1	1	1
Gh_D06G1303	No		1	1	0	0	1
Gh_D06G1588	No		0	1	1	0	1
Gh_D06G1590	No		0	1	1	1	1
Gh_D06G1591	No		1	1	1	0	1
Gh_D06G1592	Yes	*GhCRK23*	1	1	1	1	1
Gh_D06G1594	Yes	*GhCRK24*	1	1	1	1	1
Gh_D06G2195	No		1	1	1	0	1
Gh_D06G2197	Yes	*GhCRK25*	1	1	1	1	1
Gh_D08G0401	No		1	1	1	0	1
Gh_D09G0666	Yes	*GhCRK26*	1	1	1	1	1
Gh_D09G0676	No		0	1	1	1	1
Gh_D09G0677	Yes	*GhCRK27*	1	1	1	1	1
Gh_D09G0678	Yes	*GhCRK28*	1	1	1	1	1
Gh_D09G0680	No		0	1	0	1	1
Gh_D09G0682	Yes	*GhCRK29*	1	1	1	1	1
Gh_D09G1621	No		0	1	1	1	1
Gh_D10G0156	Yes	*GhCRK30*	1	1	1	1	1
Gh_D10G0157	Yes	*GhCRK31*	1	1	1	1	1
Gh_D10G0161	No		0	1	1	1	1
Gh_D10G0162	Yes	*GhCRK32*	1	1	1	1	1
Gh_D10G0163	Yes	*GhCRK33*	1	1	1	1	1
Gh_D10G0840	Yes	*GhCRK34*	1	1	1	1	1
Gh_D10G0841	Yes	*GhCRK35*	1	1	1	1	1
Gh_D11G0005	No		1	1	0	1	1
Gh_D12G1453	Yes	*GhCRK36*	1	1	1	1	1
Gh_D12G1454	Yes	*GhCRK37*	1	1	1	1	1
Gossypium arboreum							
Cotton_A_00100_BGI-A2_v1.0	Yes	*GaCRK01*	1	1	1	1	1
Cotton_A_04709_BGI-A2_v1.0	No		0	1	1	1	1
Cotton_A_07641_BGI-A2_v1.0	Yes	*GaCRK02*	1	1	1	1	1

Candidate gene ID	Classical CRK gene	Alias name	Signal peptide domain	DUF26-1 domain	DUF26-2 domain	Transmembrane domain	Kinase domain
Cotton_A_11534_BGI-A2_v1.0	No		0	1	1	1	1
Cotton_A_13085_BGI-A2_v1.0	No		0	1	1	1	1
Cotton_A_15085_BGI-A2_v1.0	No		0	1	1	1	1
Cotton_A_16175_BGI-A2_v1.0	Yes	*GaCRK03*	1	1	1	1	1
Cotton_A_16176_BGI-A2_v1.0	Yes	*GaCRK04*	1	1	1	1	1
Cotton_A_17628_BGI-A2_v1.0	Yes	*GaCRK05*	1	1	1	1	1
Cotton_A_17629_BGI-A2_v1.0	Yes	*GaCRK06*	1	1	1	1	1
Cotton_A_17636_BGI-A2_v1.0	Yes	*GaCRK07*	1	1	1	1	1
Cotton_A_20811_BGI-A2_v1.0	No		0	1	1	1	1
Cotton_A_23056_BGI-A2_v1.0	Yes	*GaCRK08*	1	1	1	1	1
Cotton_A_23058_BGI-A2_v1.0	Yes	*GaCRK09*	1	1	1	1	1
Cotton_A_30881_BGI-A2_v1.0	No		0	1	1	1	1
Cotton_A_30882_BGI-A2_v1.0	Yes	*GaCRK10*	1	1	1	1	1
Cotton_A_30883_BGI-A2_v1.0	Yes	*GaCRK11*	1	1	1	1	1
Cotton_A_30885_BGI-A2_v1.0	Yes	*GaCRK12*	1	1	1	1	1
Cotton_A_30891_BGI-A2_v1.0	No		0	1	0	1	1
Cotton_A_33667_BGI-A2_v1.0	No		0	1	1	1	1
Cotton_A_33668_BGI-A2_v1.0	Yes	*GaCRK13*	1	1	1	1	1
Cotton_A_34733_BGI-A2_v1.0	Yes	*GaCRK14*	1	1	1	1	1
Cotton_A_34735_BGI-A2_v1.0	Yes	*GaCRK15*	1	1	1	1	1
Cotton_A_34736_BGI-A2_v1.0	Yes	*GaCRK16*	1	1	1	1	1
Cotton_A_35609_BGI-A2_v1.0	Yes	*GaCRK17*	1	1	1	1	1
Cotton_A_35690_BGI-A2_v1.0	No		1	1	1	2	1
Cotton_A_36143_BGI-A2_v1.0	No		0	1	0	1	1
Cotton_A_38975_BGI-A2_v1.0	No		0	1	1	0	1
Cotton_A_39041_BGI-A2_v1.0	Yes	*GaCRK18*	1	1	1	1	1
Cotton_A_39042_BGI-A2_v1.0	Yes	*GaCRK19*	1	1	1	1	1
Gossypium raimondii							
Cotton_D_gene_10001004	Yes	*GrCRK01*	1	1	1	1	1
Cotton_D_gene_10001005	Yes	*GrCRK02*	1	1	1	1	1
Cotton_D_gene_10003154	No		0	1	0	1	1
Cotton_D_gene_10003188	No		1	1	1	0	1
Cotton_D_gene_10004045	No		1	1	1	0	1
Cotton_D_gene_10005630	No		0	1	1	1	1
Cotton_D_gene_10005632	Yes	*GrCRK03*	1	1	1	1	1
Cotton_D_gene_10006109	Yes	*GrCRK04*	1	1	1	1	1
Cotton_D_gene_10006110	Yes	*GrCRK05*	1	1	1	1	1

Candidate gene ID	Classical CRK gene	Alias name	Signal peptide domain	DUF26-1 domain	DUF26-2 domain	Transmembrane domain	Kinase domain
Cotton_D_gene_10007069	Yes	*GrCRK06*	1	1	1	1	1
Cotton_D_gene_10008978	Yes	*GrCRK07*	1	1	1	1	1
Cotton_D_gene_10010032	No		0	1	1	1	1
Cotton_D_gene_10012432	No		0	1	0	1	1
Cotton_D_gene_10017936	Yes	*GrCRK08*	1	1	1	1	1
Cotton_D_gene_10018038	Yes	*GrCRK09*	1	1	1	1	1
Cotton_D_gene_10018766	Yes	*GrCRK10*	1	1	1	1	1
Cotton_D_gene_10019420	No		0	1	1	1	1
Cotton_D_gene_10020588	No		0	1	1	1	1
Cotton_D_gene_10020589	Yes	*GrCRK11*	1	1	1	1	1
Cotton_D_gene_10020593	Yes	*GrCRK12*	1	1	1	1	1
Cotton_D_gene_10020595	Yes	*GrCRK13*	1	1	1	1	1
Cotton_D_gene_10020599	No		0	1	0	1	1
Cotton_D_gene_10022247	No		1	1	1	0	1
Cotton_D_gene_10022248	No		1	1	1	0	1
Cotton_D_gene_10022250	No		1	1	0	0	1
Cotton_D_gene_10022252	No		0	1	1	0	1
Cotton_D_gene_10023172	Yes	*GrCRK14*	1	1	1	1	1
Cotton_D_gene_10031260	No		0	1	1	1	1
Cotton_D_gene_10031261	Yes	*GrCRK15*	1	1	1	1	1
Cotton_D_gene_10031262	No		1	1	1	0	1
Cotton_D_gene_10031263	No		0	1	1	0	1
Cotton_D_gene_10031265	Yes	*GrCRK16*	1	1	1	1	1
Cotton_D_gene_10031266	Yes	*GrCRK17*	1	1	1	1	1
Cotton_D_gene_10031267	No		0	1	1	1	1
Cotton_D_gene_10032380	No		1	1	1	0	1
Cotton_D_gene_10033817	No		0	1	1	1	1
Cotton_D_gene_10034823	Yes	*GrCRK18*	1	1	1	1	1
Cotton_D_gene_10034824	No		0	1	1	1	1
Cotton_D_gene_10035010	No		0	1	0	1	1
Cotton_D_gene_10035014	Yes	*GrCRK19*	1	1	1	1	1
Cotton_D_gene_10040971	Yes	*GrCRK20*	1	1	1	1	1
Cotton_D_gene_10040972	Yes	*GrCRK21*	1	1	1	1	1
Cotton_D_gene_10040974	No		1	1	1	0	1
Arabidopsis thaliana							
AT1G19090.1	Yes	*AtCRK01*	1	1	1	1	1
AT1G70520.1	Yes	*AtCRK02*	1	1	1	1	1

Candidate gene ID	Classical CRK gene	Alias name	Signal peptide domain	DUF26-1 domain	DUF26-2 domain	Transmembrane domain	Kinase domain
AT1G70530.1	Yes	*AtCRK03*	1	1	1	1	1
AT3G45860.1	Yes	*AtCRK04*	1	1	1	1	1
AT4G00970.1	Yes	*AtCRK05*	1	1	1	1	1
AT4G04490.1	Yes	*AtCRK06*	1	1	1	1	1
AT4G04500.1	Yes	*AtCRK07*	1	1	1	1	1
AT4G04510.1	Yes	*AtCRK08*	1	1	1	1	1
AT4G04540.1	Yes	*AtCRK09*	1	1	1	1	1
AT4G04570.1	Yes	*AtCRK10*	1	1	1	1	1
AT4G05200.1	Yes	*AtCRK11*	1	1	1	1	1
AT4G11460.1	Yes	*AtCRK12*	1	1	1	1	1
AT4G11470.1	Yes	*AtCRK13*	1	1	1	1	1
AT4G11480.1	Yes	*AtCRK14*	1	1	1	1	1
AT4G11490.1	Yes	*AtCRK15*	1	1	1	1	1
AT4G11530.1	Yes	*AtCRK16*	1	1	1	1	1
AT4G21230.1	Yes	*AtCRK17*	1	1	1	1	1
AT4G21400.1	Yes	*AtCRK18*	1	1	1	1	1
AT4G21410.1	Yes	*AtCRK19*	1	1	1	1	1
AT4G23130.1	Yes	*AtCRK20*	1	1	1	1	1
AT4G23140.1	Yes	*AtCRK21*	1	1	1	1	1
AT4G23150.1	Yes	*AtCRK22*	1	1	1	1	1
AT4G23160.1	No		0	1	1	1	1
AT4G23180.1	Yes	*AtCRK23*	1	1	1	1	1
AT4G23190.1	Yes	*AtCRK24*	1	1	1	1	1
AT4G23200.1	No		1	1	1	0	1
AT4G23210.1	Yes	*AtCRK25*	1	1	1	1	1
AT4G23220.1	No		0	1	1	1	1
AT4G23230.1	No		0	1	0	1	1
AT4G23250.1	Yes	*AtCRK26*	1	1	1	1	1
AT4G23260.1	Yes	*AtCRK27*	1	1	1	1	1
AT4G23270.1	Yes	*AtCRK28*	1	1	1	1	1
AT4G23280.1	Yes	*AtCRK29*	1	1	1	1	1
AT4G23290.1	No		0	1	1	1	1
AT4G23300.1	Yes	*AtCRK30*	1	1	1	1	1
AT4G23320.1	No		0	0	0	1	1
AT4G28670.1	Yes	*AtCRK31*	1	1	1	1	1
AT4G38830.1	Yes	*AtCRK32*	1	1	1	1	1
AT5G40380.1	Yes	*AtCRK33*	1	1	1	1	1

Candidate gene ID	Classical CRK gene	Alias name	Signal peptide domain	DUF26-1 domain	DUF26-2 domain	Transmembrane domain	Kinase domain
Oryza sativa							
Os01g0342200	No		1	1	1	0	1
Os01g0366300	No		0	1	1	1	1
Os01g0568400	Yes	*OsCRK01*	1	1	1	1	1
Os05g0493100	Yes	*OsCRK02*	1	1	1	1	1
Os07g0487400	No		1	1	0	1	1
Os07g0534700	Yes	*OsCRK03*	1	1	1	1	1
Os07g0535800	Yes	*OsCRK04*	1	1	1	1	1
Os07g0537000	Yes	*OsCRK05*	1	1	1	1	1
Os07g0537500	Yes	*OsCRK06*	1	1	1	1	1
Os07g0537900	Yes	*OsCRK07*	1	1	1	1	1
Os07g0538200	No		0	1	1	1	1
Os07g0540100	Yes	*OsCRK08*	1	1	1	1	1
Os07g0540800	Yes	*OsCRK09*	1	1	1	1	1
Os07g0541000	Yes	*OsCRK10*	1	1	1	1	1
Os07g0541400	Yes	*OsCRK11*	1	1	1	1	1
Os07g0541500	Yes	*OsCRK12*	1	1	1	1	1
Os07g0541800	Yes	*OsCRK13*	1	1	1	1	1
Os07g0541900	Yes	*OsCRK14*	1	1	1	1	1
Os07g0542400	Yes	*OsCRK15*	1	1	1	1	1
Os07g0628700	Yes	*OsCRK16*	1	1	1	1	1
Os07g0628900	Yes	*OsCRK17*	1	1	1	1	1
Os10g0136400	Yes	*OsCRK18*	1	1	1	1	1
Os10g0136500	No		0	1	1	1	1
Os10g0327000	No		1	1	1	0	1
Os11g0601500	Yes	*OsCRK19*	1	1	1	1	1
Os11g0681600	No		1	1	1	0	1
Os12g0606000	Yes	*OsCRK20*	1	1	1	1	1
Os12g0608500	Yes	*OsCRK21*	1	1	1	1	1
Os12g0608700	No		0	1	1	1	1
Os12g0608900	Yes	*OsCRK22*	1	1	1	1	1
Zea mays							
NP_001140883.1	No		0	1	1	1	1
NP_001142138.1	No		0	1	0	1	1
NP_001145947.1	Yes	*ZmCRK01*	1	1	1	1	1
NP_001146379.1	Yes	*ZmCRK02*	1	1	1	1	1

Candidate gene ID	Classical CRK gene	Alias name	Signal peptide domain	DUF26-1 domain	DUF26-2 domain	Transmembrane domain	Kinase domain
NP_001146710.1	Yes	*ZmCRK03*	1	1	1	1	1
NP_001147287.1	Yes	*ZmCRK04*	1	1	1	1	1
NP_001147825.1	Yes	*ZmCRK05*	1	1	1	1	1
NP_001152742.1	Yes	*ZmCRK06*	1	1	1	1	1
XP_008648512.1	Yes	*ZmCRK07*	1	1	1	1	1
XP_008649610.1	Yes	*ZmCRK08*	1	1	1	1	1
XP_008649611.1	Yes	*ZmCRK09*	1	1	1	1	1
XP_008649810.1	Yes	*ZmCRK10*	1	1	1	1	1
XP_008651081.1	Yes	*ZmCRK11*	1	1	1	1	1
XP_008651082.1	Yes	*ZmCRK12*	1	1	1	1	1
XP_008651129.1	Yes	*ZmCRK13*	1	1	1	1	1
XP_008651392.2	No		0	1	1	1	1
XP_008652974.1	Yes	*ZmCRK14*	1	1	1	1	1
XP_008653173.1	Yes	*ZmCRK15*	1	1	1	1	1
XP_008653174.1	Yes	*ZmCRK16*	1	1	1	1	1
XP_008653175.1	Yes	*ZmCRK17*	1	1	1	1	1
XP_008653190.2	No		0	1	1	1	1
XP_008655309.2	Yes	*ZmCRK18*	1	1	1	1	1
XP_008656588.1	Yes	*ZmCRK19*	1	1	1	1	1
XP_008657186.3	Yes	*ZmCRK20*	1	1	1	1	1
XP_008660731.1	Yes	*ZmCRK21*	1	1	1	1	1
XP_008661993.1	Yes	*ZmCRK22*	1	1	1	1	1
XP_008663149.1	No		0	1	1	1	1
XP_008663152.1	No		0	1	1	1	1
XP_008667806.1	Yes	*ZmCRK23*	1	1	1	1	1
XP_008668297.1	Yes	*ZmCRK24*	1	1	1	1	1
XP_008670468.1	Yes	*ZmCRK25*	1	1	1	1	1
XP_008670469.1	No		1	1	1	0	1
XP_008674038.1	Yes	*ZmCRK26*	1	1	1	1	1
XP_008674707.1	Yes	*ZmCRK27*	1	1	1	1	1
XP_008676391.1	Yes	*ZmCRK28*	1	1	1	1	1
XP_008680980.1	Yes	*ZmCRK29*	1	1	1	1	1
XP_008680981.1	Yes	*ZmCRK30*	1	1	1	1	1
XP_020394856.1	No		1	1	1	0	1
XP_020394857.1	No		1	1	1	0	1
XP_020395137.1	Yes	*ZmCRK31*	1	1	1	1	1
XP_020395138.1	Yes	*ZmCRK32*	1	1	1	1	1
XP_020395139.1	Yes	*ZmCRK33*	1	1	1	1	1

Candidate gene ID	Classical CRK gene	Alias name	Signal peptide domain	DUF26-1 domain	DUF26-2 domain	Transmembrane domain	Kinase domain
XP_020395142.1	Yes	*ZmCRK34*	1	1	1	1	1
XP_020395143.1	Yes	*ZmCRK35*	1	1	1	1	1
XP_020395144.1	Yes	*ZmCRK36*	1	1	1	1	1
XP_020402049.1	No		0	1	1	1	1
XP_020402050.1	No		0	1	1	0	1
XP_020404164.1	No		0	1	1	1	1
XP_020404165.1	Yes	*ZmCRK37*	1	1	1	1	1
XP_020404772.1	Yes	*ZmCRK38*	1	1	1	1	1
XP_023156181.1	No		0	1	1	1	1
