# Supplementary material for: Genome-Wide Identification and Functional Analyses of the CRK Gene Family in Cotton Reveals GbCRK18 Confers Verticillium Wilt Resistance in Gossypium barbadense
Source: Front Plant Sci. 2018 Sep 11;9:1266. doi: 10.3389/fpls.2018.01266 (PMC6141769; doi:10.3389/fpls.2018.01266)
Supplement: Supplementary file 4 [file Image_1.pdf]

Supplementary Figures

Genome-Wide Identification and Functional Analyses of the CRK Gene Family in Cotton Reveals *GbCRK18* Confers Verticillium Wilt Resistance in *Gossypium barbadense*

Authors:

Ting-Gang Li[1,*], Dan-Dan Zhang[1,*], Lei Zhou[1], Zhi-Qiang Kong[1], Adamu, S. Hussaini[1], Dan Wang[1], Jun-Jiao Li[1], Dylan P. G. Short[2], Nikhilesh Dhar[2], Steven J. Klosterman[3], Bao-Li Wang[1], Chun-Mei Yin[1], Krishna V. Subbarao[2,#], Jie-Yin Chen[1,#], Xiao-Feng Dai[1,#]

[*]These authors contributed equally to this work

Institutional affiliation:

[1] Laboratory of Cotton Disease, Institute of Food Science and Technology, Chinese Academy of Agricultural Sciences, Beijing, 100193, China

[2] Department of Plant Pathology, University of California, Davis, c/o U.S. Agricultural Research Station, Salinas, California, United States of America

[3] United States Department of Agriculture, Agricultural Research Service, Crop Improvement and Protection Research Unit, Salinas, CA, USA

Correspondence:

[1] Xiao-Feng Dai and Jie-Yin Chen

The Institute of Food Science and Technology

Chinese Academy of Agricultural Sciences

Beijing 100193, P.R. China

Fax: +86-10-62813566;

E-mail: daixiaofeng_caas@126.com, chenjieyin@caas.cn;

[2] Krishna V. Subbarao

E-mail: kvsubbarao@ucdavis.edu

Running title:

CRKs involve in VW resistance

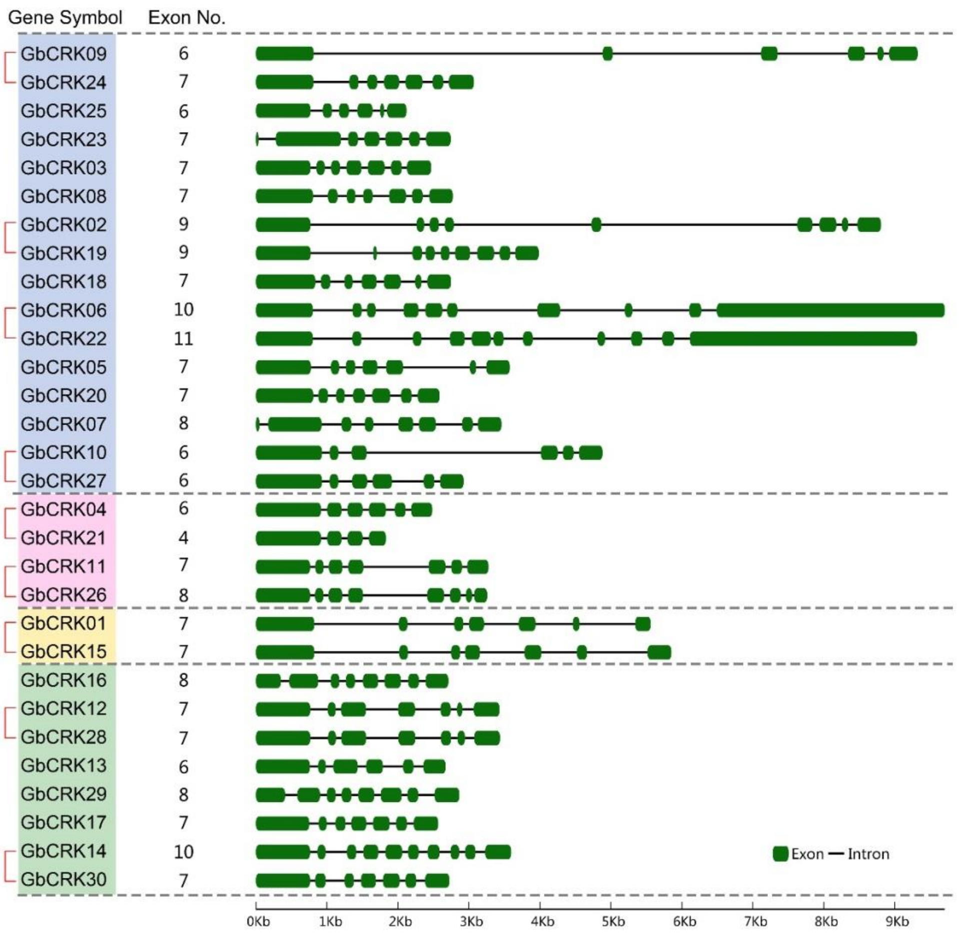

Figure S1 | Schematic for *CRK* gene intron/exon structure in *Gossypium barbadense*. The black lines indicate introns, the green boxes represent exons. Genes are listed arranged in the order they appear on the phylogenetic tree and the number of exons per gene is listed on the right of gene symbol.

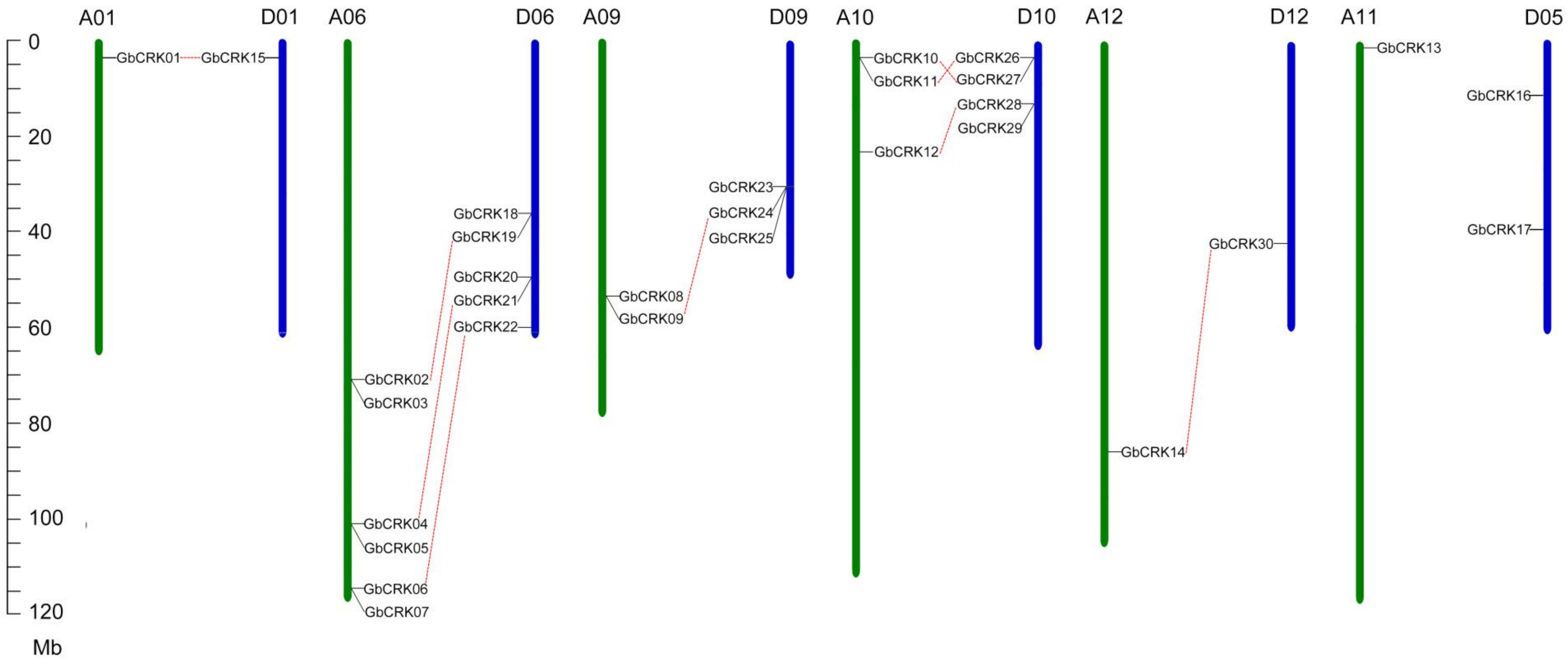

Figure S2 | Location of *CRK* genes on *Gossypium barbadense* chromosomes. Paralogous pairs of genes are linked by red dotted lines. Chromosome numbers are located at the top of each vertical bar. The scale on the left represents the lengths of the chromosomes in megabases (Mb).

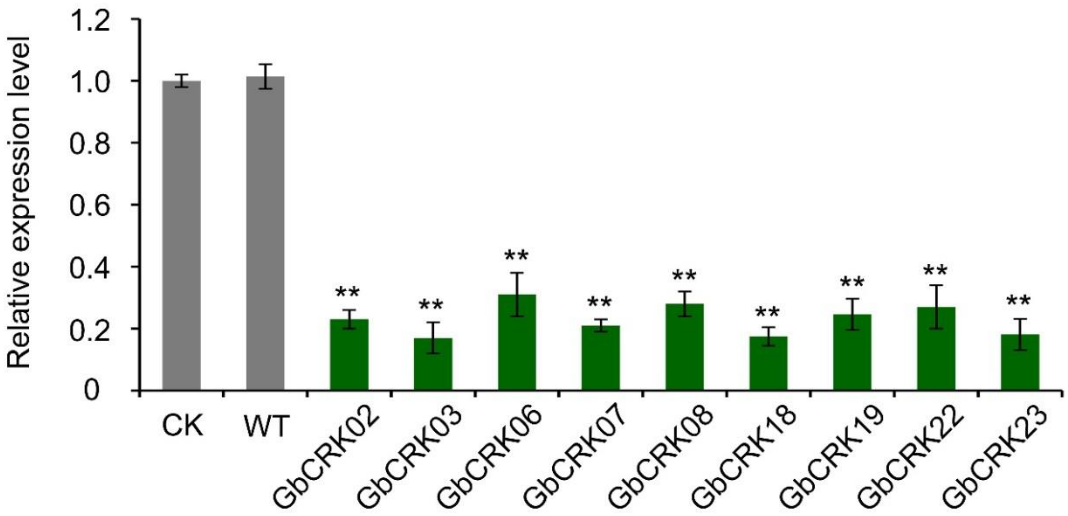

Figure S3 | Expression of *GbCRK02, GbCRK03, GbCRK06, GbCRK07, GbCRK08, GbCRK18, GbCRK19, GbCRK22* and *GbCRK23* genes in control and silenced plants as determined by reverse transcription-quantitative PCR analysis. WT and CK represent cv. Hai 7124 and pTRV2:00 respectively. Error bars were calculated based on three biological replicates using standard deviation. ** indicates significant difference ($P < 0.01$).
